# Not simply two sides of the same coin: Target enhancement and distractor suppression draw on independent neural mechanisms

**DOI:** 10.3758/s13414-025-03196-2

**Published:** 2026-04-06

**Authors:** Natalia Khodayari, Howard Egeth, Susan M. Courtney

**Affiliations:** 1 Department of Psychological and Brain Sciences, Johns Hopkins University, 3400 North Charles St, Ames Hall, Baltimore, MD 21218 USA; 2 Department of Neuroscience, Johns Hopkins University, Baltimore, MD USA

**Keywords:** Selective attention, Individual differences, Singleton search, Target enhancement, Distractor suppression, Reliability, Statistical learning

## Abstract

Enhancement and suppression have traditionally been described as a shared process; enhancing relevant information subsequently suppresses irrelevant information, but there may also exist separable mechanisms. Recent evidence indicates that target enhancement and distractor suppression can be measured separately, using both behavioral and physiological approaches. It is unclear, however, whether the underlying mechanisms are also separable. We used an individual differences approach to investigate whether enhancement and suppression strengths vary independently across individuals, which would provide strong evidence supporting the existence of separate mechanisms. This approach, however, requires dissociable, internally reliable individual-difference measures. We found reliable enhancement measures for both endogenous cueing effects (Experiment 1) and statistical learning effects (Experiment 2) for target location. Measures of suppression in Experiments 1 and 2 were unreliable. In Experiment 3, we encouraged a single strategy (“singleton-search mode”) by changing the colors and shapes of targets and distractors across trials. We found reliable individual differences in reaction time measures for enhancement and suppression. Critically, these reliable measures did not covary. Experiments 4 and 5 largely replicated the results of Experiment 3. Again, individual-difference measures of enhancement and suppression strength did not covary. These results show a clear dissociation: While enhancement yielded reliable individual differences across task manipulations, suppression measures were usually unreliable and sensitive to task parameters. When individual-difference measures of both enhancement and suppression were reliable, they did not covary. These results cannot be explained solely by a shared-mechanism account of enhancement and suppression, and instead support the hypothesis that independent enhancement and suppression mechanisms exist.

**Supplementary Information:**

The online version contains supplementary material available at 10.3758/s13414-025-03196-2.

The concept of “selective attention” has always included both enhanced processing of attended information and reduced processing of unattended (typically irrelevant or distracting) information (James, 1890). The mechanistic relationships between these two aspects of attention, however, remain unclear. Influential models of attention, such as the biased competition model (Desimone & Duncan, 1995) and the normalization model of attention (Reynolds & Heeger, 2009) focus on interdependent accounts of enhancement and suppression. Desimone and Duncan (1995) describe their model of biased competition as a process in which the activity of neurons representing relevant information can be selectively enhanced by either goal-directed top-down control or bottom-up saliency. These enhanced activity patterns then directly reduce activity of neurons that are selective for competing information (e.g., via lateral inhibition). In the normalization model of attention, enhancement effects of selective attention are multiplicative and suppression effects are divisive. Akin to the biased competition model, Reynolds and Heeger (1995) model suppression as a direct consequence of, and mathematically a function of, attentional gain (enhancement). In support of these models, research using electrophysiological recordings in nonhuman animals has demonstrated that population codes increase their selectivity (improve signal-to-noise) for target stimuli, with a corresponding decrease in neural activity coding for irrelevant, distracting, nearby stimuli (Moore & Zirnsak, 2017). Data such as these suggest a single, reciprocal mechanism that, in principle, could underlie suppression of many kinds of unattended, irrelevant information at multiple levels of the processing hierarchy, including salient distracting stimuli (Cosman et al., 2018; for reviews, see Buschman and Kastner, 2015; Carrasco, 2011). For example, if distractors appear in a different location from targets, then enhancing the expected target location could also directly suppress processing of stimuli at all nontarget locations. Similarly, if targets are consistently green and distractors are never green, for example, then enhancing the processing of green stimuli could directly result in suppressed processing of stimuli that are any color other than green.

Most attention research has focused on the enhancement of targets. However, some studies have also explored ways in which nontargets may be suppressed. Notably, Hillyard and colleagues were among the first to study the separability of enhancement and suppression mechanisms (Hillyard et al., 1998). Studies have since focused on developing means for measuring enhancement and suppression separately (e.g., Chang & Egeth, 2019; Cunningham & Egeth, 2016; van Moorselaar & Slagter, 2019; Wöstmann et al., 2019) which further hint at the possibility of separable mechanisms. Studies utilizing physiological approaches have suggested that an ERP component, known as the N2pc, is associated with stimulus enhancement while another ERP component, usually referred to as the PD, is associated with suppression of a distractor, and not with selection of targets (Carlisle & Woodman, 2011; Drisdelle & Eimer, 2021; Duncan et al., 2023; Hilimire et al., 2011; Jannati et al., 2013; McDonald et al., 2013). These physiological measures seem to have somewhat different time courses and scalp topographies (e.g., Hilimire et al., 2010, Zhao et al., 2023), but what cognitive and neural processes these signals reflect, and whether they are independent of each other, is still a matter of debate. For example, Hickey et al. (2009) describes the N2pc ERP component as a combination of enhanced target processing (NT, or the Target Negativity) and suppressed distractor processing (PD, or the Distractor Positivity). On the other hand, Sawaki and Luck (2010) found that irrelevant stimuli produced a PD component, but did not produce an N2pc component, indicating these measures can be evoked independently. In addition to ERPs, Wöstmann et al. (2019) examined evidence of functional separability from oscillatory activity in the alpha frequency range (8–12 Hz), demonstrating that alpha lateralization can be independently measured for target selection and distractor suppression with somewhat distinct scalp topographies.

Given the uncertainty about the interpretation of electrophysiological indices in answering the separability question, and without clear validation of Hillyard and colleagues’ original claim, we sought to explore an alternative approach. If selective attention utilizes only shared or interdependent mechanisms of enhancement and suppression, then individual differences in the degree to which targets are enhanced should correlate with individual differences in the degree to which distractors are suppressed. Alternatively, a lack of covariance would suggest the existence of separable mechanisms of enhancement and suppression. Here we present strong support in favor of the latter case. We used an individual differences approach to investigate whether enhancement and suppression strength vary independently across individuals. This direct approach, however, requires internally consistent (which we refer to as “internally reliable”) measures of individual differences where behavior is consistent across trials within an experimental session. Reliability of individual-difference measures has been a problem when using tasks that were initially developed to maximize differences between experimental conditions that are consistent across individuals in a group, as this has the effect of minimizing individual differences. Hedge et al. (2018) have described this issue as the “reliability paradox.” They examined test–retest reliability across a large number of tasks frequently used in psychology and cognitive neuroscience research (Eriksen Flanker, Stroop, go/no-go, stop-signal, Posner cueing, Spatial-Numerical Association of Response Codes [SNARC], Navon). These tasks have produced highly replicable between-condition effects. but mostly produced unreliable test–retest measures. In view of this, we sought to develop a task that yielded the expected between-condition effects, but also internally reliable individual differences in order to test for correlations between individual-difference measures of enhancement and those of suppression. Given internally reliable measures, different degrees of enhancement versus suppression across individuals would suggest differences in the underlying neural mechanisms. Furthermore, we also compared how internal reliability of enhancement differs from internal reliability of suppression. Differences between internal reliabilities would provide converging evidence for differences in the underlying neural mechanisms.

To foreshadow our findings, the results of four experiments, collectively, provide convergent evidence that enhancement and suppression draw on separable mechanisms. In Experiments 1 and 2, target enhancement (in Experiment 1 via endogenous cueing; in Experiment 2 via statistical learning) yielded internally reliable individual differences in reaction time (RT) and accuracy measures while suppression measures (in Experiments 1 and 2 via statistical learning) were unreliable. In Experiment 3 (via statistical learning task with constrained search strategy), we found internally reliable individual differences in RT measures for both enhancement and suppression with no significant correlation between them. Experiments 4 and 5 included a larger number of participants with relaxed performance criteria. The results of Experiments 4 and 5 largely replicated those of Experiment 3. We again found a lack of correlation between enhancement and suppression. Our contrasting results of internal reliability in Experiments 1–5 suggest that target enhancement and distractor suppression are qualitatively different: enhancement is internally reliable across cue-based and experience-based attention manipulations, while suppression reliability varies across task parameters. Further, in Experiments 3, 4, and 5, which did show internally reliable suppression measures, we found a lack of covariance between the magnitude of enhancement strength and that of suppression strength. Together, these results suggest that shared mechanisms alone cannot explain variability in behavior across individuals. Instead, our findings support the notion that mechanisms underlying distractor suppression are at least to some degree independent of those underlying target enhancement.

## Experiment 1

### Methods

#### Participants

Fifty-eight undergraduate students at Johns Hopkins University participated online in this experiment for course extra credit. Participants provided informed consent, and the protocol was approved by the Johns Hopkins Homewood Institutional Review Board. Participants were dismissed if they met any of the following criteria: (1) Mean accuracy fell below 70% across two consecutive blocks, (2) Mean RT fell outside of 250–1,500 ms across two consecutive blocks, or (3) Otherwise failed to follow instructions.

Of the 58 participants who participated, data from 12 participants were excluded from analyses due to incomplete experimental sessions. Of the remaining 46 participants, data from one participant were excluded due to reporting not having normal or corrected-to-normal vision. Thus, data from 45 participants (mean age: 19.7 [18–23]; sex: 32 women) were retained for analysis.

#### Apparatus

The experiment was written in JavaScript using the psiTurk framework (Gureckis et al., 2016), CSS, and HTML for development of the task. Python was used for deployment to the cloud server Heroku with GitHub integration. Data were collected continuously using the Heroku Postgres database. Stimuli were designed on GIMP (Version 2.10; The GIMP Development Team, 2020). The experiment was conducted online using participants’ personal laptop or desktop computers. Participants were instructed to adhere to a viewing distance of approximately 2 ft (60.69 cm) from the center of their computer screen, sit in an upright position, and use their keyboard to respond to the target using their right hand. Participants responded to the target by pressing either the down arrow key using their index finger, or the right arrow key using their middle finger.

All instructions were provided in written format. Although experiment instructions were designed to minimize variability in experimental conditions such as seating distance, monitor display settings, and so forth, because the study was run online, it was not possible to control environmental conditions. Visual angles (based on the nominal viewing distance of 60.69 cm) and RGB values are detailed below for replication purposes.

#### Stimuli and task design

**Fig. 1:**
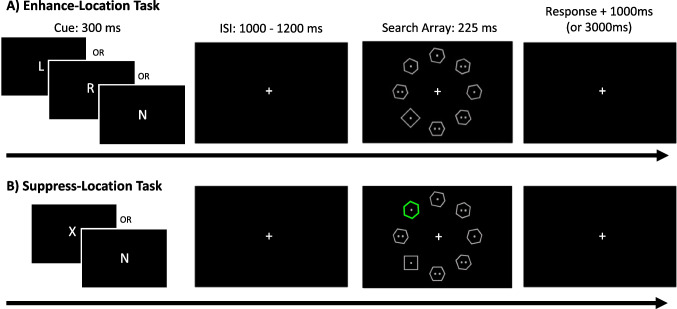
Experiment 1 Design. **A**) Enhance-Location task. Participants were presented with a letter cue which explicitly informed them of the likely location of the target ("L" for left side, "R" for right side, ''N" for non-informative). Following a jittered delay, the target array appeared. Participants reported the number of dots (one or two) inside the target shape (square or diamond) as quickly and accurately as possible. **B**) Suppress-Location task. Same goal and structure as in Enhance-Location, but instead of explicit cues, participants learned from experience (statistical learning) the most-probable distractor (colored hexagon) location. Letter cues used here held no information pertaining to the likely location of the distractor. Ninety percent of trials used "X" cues indicating a distractor was present. Ten percent of trials used an "N" cue indicating the distractor was absent

Our tasks (adapted from Posner, 1980; Wang & Theeuwes, 2018b) involved searching for a singleton shape within a circular array of shapes that were otherwise all identical to each other and different from the target. All stimuli were presented on a black background (see Fig. 1). A center fixation cross (~0.75° × 0.75°) was present throughout each trial. A trial began with a letter cue (~1.00 ^o^ × 1.00°, described later) presented at fixation for 300 ms. This was followed by a delay period of 1,000–1,200 ms (pseudorandomized in 100-ms increments). Following the delay period, an eight-item circular search array (visual angle radius of ~3.19°, from the fixation to the center of each display element) was presented for 225 ms. The search array contained one target shape—square (~1.32° × 1.32°) or diamond (~1.79° × 1.79°)—and seven nontarget hexagons (~ 1.60° x 1.60°). Each nontarget hexagon was randomly rotated 0°, 10°, 20°, 30°, 40°, 50°, or 60° (clockwise or counterclockwise) to reduce unwanted effects of nontargets looking more similar to one versus the other of the two target shapes. All shapes were grey outlines (unless one of the hexagons was a distractor color-singleton, as described below). Each shape contained either one or two white dots. Participants were to report the number of dots (one or two) inside the target shape (either a square or diamond, selected randomly on each trial) as quickly and accurately as possible. The trial ended after either response via button press or 2225 ms (after array-onset) if there was no response. The next trial began 1000 ms after the end of the previous trial (inter-trial-interval). Participants were instructed to maintain fixation at the central fixation cross during the entirety of each trial. Participants were instructed to use the letter cue, presented at the beginning of each trial, to prepare for the upcoming search array.

Experiment 1 consisted of four tasks in separate blocks of trials: Enhance-Location, Enhance-Feature, Suppress-Location, and Suppress-Feature. The enhancement tasks (Enhance-Location, Enhance-Feature) were adapted from Posner (1980) and the suppression tasks (Suppress-Location, Suppress-Feature) from Wang and Theeuwes (2018b). There were two blocks of trials for each task, resulting in a total of eight blocks. The current paper includes only results from the Enhance-Location and Suppress-Location blocks. Please see Supplemental Materials Section 1.1.1. for details regarding methods for the Enhance-Feature and Suppress-Feature blocks. Results from those blocks will be reported separately.

During the Enhance-Location task, participants were cued with a letter to the likely location of the target, “R” for right or “L” for left side of the array, with equal probability. These cues validly predicted the target side on 70% of trials (valid trials) and invalidly predicted the target side on 20% of trials (invalid trials). The “N” letter cue (present on 10% of trials) did not provide any information about the upcoming target location (non-informative trials). Thus, of the 160 total trials for Enhance-Location (80 trials per block), each participant completed 112 valid trials, 32 invalid trials, and 16 non-informative trials. Targets never appeared at the two vertical meridian locations (12 o’clock and 6 o’clock positions), as our cues were confined to the right and left sides only. The Enhance-Location task did not include any distractor stimuli.

During the Suppress-Location task, participants could learn through experience (via statistical learning) the likely location of the distractor singleton. One of the nontarget hexagons served as the distractor singleton and had a unique color, randomly selected on each trial from a set of five possible colors: yellow (RGB 255, 215, 0), blue (RGB 0, 129, 255), purple (RGB 179, 0, 255), green (0, 255, 0), or magenta (RGB 255, 20, 147). A letter cue, “X” or “N,” was used to prepare participants for the upcoming search array. An “X” cue indicated a distractor would be presented in that trial (90% of Suppress-Location trials), but no information was provided regarding the upcoming distractor singleton’s location or color. The distractor would appear in one specific location of the array (frequent trials) 70% of the time and in any of the other locations (infrequent trials), at random, 20% of the time (4% of the time in each of the remaining 5 non-vertical-meridian locations); distractor singleton color was random. The target never appeared at the frequent distractor location. An “N” cue indicated that no distractor would appear in that trial (distractor-absent trials, 10% of the Suppress-Location trials). Thus, of the 160 total trials for Suppress-Location (80 trials per block), each participant completed 112 frequent trials, 32 infrequent trials, and 16 distractor-absent trials. Neither targets nor distractors appeared at the two vertical meridian locations (12 o’clock and 6 o’clock positions) to match our Enhance-Location task design.

Participants completed three practice blocks of 5 trials each, for a total of 15 trials: (1) Cueing to the target side (Enhance-Location), (2) Cueing to the target shape (Enhance-Feature), and (3) Presenting distractor-singletons (to gain practice with the presence of distractors for suppression blocks [i.e., Suppress-Location and Suppress-Feature blocks]).

If participants did not meet our performance criteria of both 80% accuracy and a mean RT within 250–1500 ms in any practice block, they would receive another practice block of five trials of the same task. Participants were provided a maximum of four practice attempts per block type (four attempts for cued location practice, four attempts for cued shape practice, and four attempts for distractor-singleton practice). Performance feedback was provided after each practice block. If a participant failed to achieve the accuracy and RT thresholds after four consecutive practice blocks they were dismissed from the study. Participants who met the speed and accuracy criteria on all practice block task types proceeded to the main task consisting of eight blocks (80 trials each): two blocks for each task. Trial blocks and trial orders were pseudorandomized. At the end of each block, participants received both performance feedback and information about the upcoming block type (which cues would be presented and brief task instructions). Participants began the next block when they were ready. Within each block, an automatic break lasting 10 s appeared after each third of the trials (~27 trials) was completed, providing participants two short breaks to briefly rest and prepare for the remainder of the block.

Following completion of the task blocks, participants completed a survey comprised of demographic questions and a series of questionnaires (see Supplemental Materials Section 1.1.2). The entire experiment took roughly one hour to complete.

#### Analysis

All analyses were performed in R (Version 4.3.2; R Core Team, 2023). Experiments 1–4 used the same R packages and functions.

##### Mean differences

RT and accuracy difference scores were compared between conditions (Validity Effect: invalid target cue – valid target cue, Frequency Effect: infrequent distractor location – frequent distractor location) using Bonferroni-corrected paired *t* tests (“t.test()” function in R).^1^ For both tasks, trials with an RT outside of 250–2225 ms, no response, or inappropriate key press (any key other than the right- and down-arrow response keys) were removed from accuracy analyses (1.8%). Incorrect trials were additionally removed for RT analyses (12.6%). Mean differences were plotted using the *ggplot2* package (Version 3.4.2; Wickham, 2016).

To account for individual differences in baseline response speed and accuracy, we conducted post hoc normalized *t* tests using a difference-of-sums approach (i.e., Validity Effect): $\left(\mathrm{invalid}-\mathrm{valid}\right)/\left(\mathrm{invalid}+\mathrm{valid}\right)$, Frequency Effect:$\left(\mathrm{infrequent}-\mathrm{frequent}\right)/\left(\mathrm{infrequent}+\mathrm{frequent}\right)$:

$$\frac{{condition}_{2}-{condition}_{1}}{{condition}_{2}+{condition}_{1}}$$

For RT, normalization was performed by dividing the difference between conditions by the sum of their RT values. Accuracy was similarly normalized using the percent difference divided by the sum of percentage correct across conditions. Normalized results are presented in Table 1. For reference, the original (non-normalized) mean difference measures are also presented in the right-most column of Table 1.

**Table 1:** Experiment 1 Normalized Pairwise Comparisons

Condition	Measurement	df	t score	*p*	Mean % change ± se	*95% CI*	Invalid/Infrequent	Valid/Frequent	Original (non-normalized) mean difference ± se
Enhance Location—Validity Effect	Accuracy	44	−5.84	< .00001 *****	−11.1 **±** 0.02	[−15.0, −7.3]	76.2%	92.8%	−16.6% **±** 2.6%
Enhance Location—Validity Effect	Reaction Time	44	6.60	< .00001 *****	8.0 **±** 1.2	[5.5, 10.4]	1005 ms	844 ms	161 ms **±** 25 ms
Suppress Location—Frequency Effect	Accuracy	44	−3.98	< .001 ***	−2.1% **±** 0.5	[−3.1, −1.0]	86.7%	90.1%	−3.4% **±** 0.8%
Suppress Location—Frequency Effect	Reaction Time	44	4.96	< .0001 ****	1.8 **±** 0.4	[1.1, 2.5]	877 ms	847 ms	31 ms **±** 6 ms

Mean reaction time is rounded to the nearest 1 ms and accuracy the nearest 0.1%. *ns* not significant * *p* < .05 ** *p* < .01 *** *p* < .001 **** *p* < .0001 ***** *p* < .00001

##### Individual differences

Reliability of individual-difference measures for both the Enhance-Location and Suppress-Location tasks was measured using a Spearman–Brown corrected (r_SB_), split-half analysis (“splithalf()” function from Version 0.8.2 of the *splithalf* package in R; Parsons, 2021) with 5,000 random splits. In this method, a single split-half correlation is measured by selecting, at random, half of each participant’s trials (rather than first half versus last half split, or odd versus even split). We then estimated the full correlation coefficient (R_full_) given this half correlation (R_half_). This method was repeated 5,000 times where for each iteration a new random split was generated. The distribution of our 5000 estimates was then analyzed. There is no consensus for a standard practice of quantifying the degree of internal reliability from these measures. Some have generated their own thresholds for internal reliability, while others argue that generating thresholds may limit the benefit of this continuous scale (reviewed in Parsons et al., 2019). We defined internal reliability categorically: whether the 95% confidence interval of the distribution crossed zero (unreliable) or did not cross zero (reliable). Internal reliability plots were generated using Version 3.4.2 of the *ggplot2* package.

We planned to perform a correlation analysis between the strength of target enhancement (Validity Effect: invalid target cue – target valid cue) and the strength of distractor suppression (Frequency Effect: infrequent distractor location – frequent distractor location) if both the enhancement and suppression tasks yielded internally reliable individual-differences measures (“cor.test()” function in R). We additionally planned to run a Bayes factor analysis using the “correlationBF()” function from Version 0.9.12.4.4 of the R package *BayesFactor* (Morey & Rouder, 2022) using thresholds indicated by Van der Linden et al. (2018), originally adapted from Jeffreys (1961). Bayes factors (BF10) less than 1 indicates evidence in favor of the null hypothesis (H0) while BF10 greater than 1 indicates evidence in favor of the alternative hypothesis (HA). Correlation plots were generated using version 0.6.0 of the *ggpubr* package (Kassambara, 2018).

### Results

#### Mean differences

See Table 1.

##### Reaction time

For Enhance-Location blocks, we found a significant Validity Effect, wherein participants were faster in reporting the target during validly-cued trials than invalidly cued trials, *t*(44) = 6.60,* p* < .00001. For Suppress-Location blocks, we found a significant Frequency Effect wherein participants were faster in reporting the target when the distractor singleton appeared at its frequent location than when it appeared in an infrequent location, *t*(44) = 4.96,* p* < .0001.

##### Accuracy

Mean differences in task accuracy were consistent with the RT effects with no indication of a speed–accuracy trade-off. For Enhance-Location blocks, we found a significant Validity Effect, wherein participants were more accurate in reporting the target during validly cued trials than invalidly cued trials, *t*(44) = −5.84,* p* < .00001. For Suppress-Location blocks, we found a significant Frequency Effect wherein participants were more accurate in reporting the target when the distractor singleton appeared at its frequent location than when it appeared in an infrequent location, *t*(44) = −3.98,* p* < .001.

#### Individual differences

**Fig. 2:**
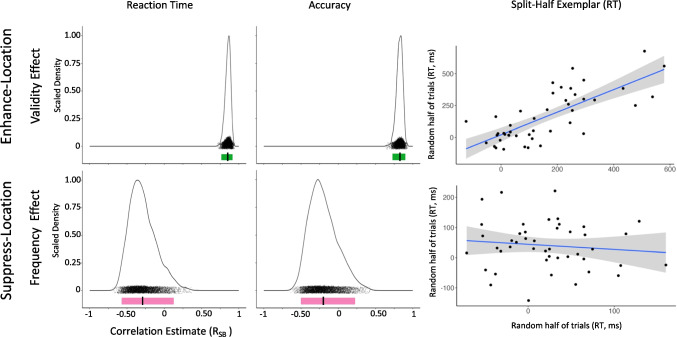
Experiment 1 density plot of Spearman Brown-corrected split half analysis with 5000 random splits for Enhance-Location (top row) and Suppress-Location (bottom row) reaction time (left column) and accuracy (middle column) individual-difference measures. Black dots underneath plot represent each of the 5000 split half correlations, jittered vertically for visualization. Colored bar (light red or dark green) below black dots represent the range of the 95% CI. If the 95% CI does not cross R_SB_ of 0 on the x-axis, the individual-difference measures are deemed reliable (dark green), otherwise it is deemed unreliable (light red). The vertical black link inside the colored bar represents the average estimate. Exemplar plots (right column) illustrate the average RT correlation estimate for a single split half for both Enhance- and Suppress-Location blocks. This is only provided to support conceptualization of the reliability estimates

See Table 2 and Fig. 2.

**Table 2:** Experiment 1 Split-Half Analyses

Condition	Measurement	n	Reliability	Mean r_SB_	95% CI
Enhance Location—Validity Effect	Accuracy	45	Reliable	.82	[.73, .89]
Enhance Location—Validity Effect	Reaction Time	45	Reliable	.85	[.77, .91]
Suppress Location—Frequency Effect	Accuracy	45	Unreliable	-.20	[-.50, .22]
Suppress Location—Frequency Effect	Reaction Time	45	Unreliable	-.29	[-.57, .12]

##### Validity effect

For Enhance-Location, split-half analyses yielded internally reliable individual-difference measures for the Validity Effect for both RT, r_SB_ = 0.85, 95% CI [0.77, 0.91], and accuracy, r_SB_ = 0.82, 95% CI [0.73, 0.89].

##### Frequency effect

Suppress-Location, however, yielded unreliable individual-difference measures for the Frequency Effect for both RT, r_SB_ = −0.29, 95% CI [−0.57, 0.12], and accuracy, r_SB_ = −0.20, 95% CI [−0.50, 0.22], suggesting that these suppression effects are not consistent within an individual across trials in our task. Due to this within-task unreliability, individual differences comparisons between enhancement and suppression measures would not be interpretable and so were not performed.

### Conclusions

Our spatial enhancement task (Enhance-Location) yielded both significant Validity Effects at the group-level, as expected, and internally reliable individual differences. These results demonstrate the utility of our enhancement task for effective group-level and individual differences analyses. There is still debate, however, whether the Validity Effect solely reflects enhancement processes. Although prior research has shown facilitation of target locations following shifts of attention from cues (Bashinski & Bacharach, 1980; Wurtz & Goldberg, 1972), it is unclear whether voluntary attention includes both facilitation of target processing and inhibition of nontarget processing **(**Couperus & Mangun, 2010). Furthermore, behavior during neutral trials has been found to be design-dependent, thus interpretation of validity effects relative to a “true” baseline is challenging (Jonides & Mack, 1984; Wöstmann et al., 2022). In contrast, studies of experience-based learning, which manipulate the spatial probability of a stimulus and is distinct from priming effects (Druker & Anderson, 2010), provide replicated evidence of target-specific enhancement (Geng & Behrmann, 2005; Zhang et al., 2022) as well as distractor-specific suppression (Wang & Theeuwes, 2018a, b; Zhang et al., 2019). For these reasons, our following experiments utilize a single statistical learning design for both target enhancement and distractor suppression measures.

Our spatial suppression task (Suppress-Location) yielded significant Frequency Effects at the group-level but individual differences in this effect were not internally reliable. This unreliability might be telling us something about the nature of suppression mechanisms. Then again, this unreliability might be reflective of something particular to the statistical learning paradigm (e.g., within-participant fluctuations during statistical learning, perhaps from changes in learning rate or task strategies). This was investigated in Experiment 2, where we examined whether statistical learning of target enhancement also yields unreliable individual-difference measures.

## Experiment 2

Experiment 2 examined whether the use of statistical learning methodology may have been responsible for the lack of internal reliability of individual-difference measures of suppression in Experiment 1. If the use of statistical learning is responsible, then if target enhancement and distractor suppression were both evaluated in a statistical learning paradigm, we might expect that both would yield unreliable individual-difference measures. Thus, in Experiment 2, we used a single statistical learning task to examine the internal reliability of individual differences in both target enhancement and distractor suppression. Unlike Experiment 1, in Experiment 2 distractors were present on some trials during both our blocks manipulating target enhancement (i.e., Target-Location Learning [TLL] blocks; see Table 3 for glossary of naming conventions) and our blocks manipulating distractor suppression (i.e., Distractor-Location Learning [DLL] blocks). By including distractors in both block types, we can examine whether the same measure of distractor capture (distractor present – distractor absent) correlates across blocks. A lack of correlation between measures of distractor capture would suggest that noise generated from state changes (e.g., motivation) may weaken the observed strength of true correlations across blocks. Thus, these changes in Experiment 2 enabled us to (1) maintain design consistency across both block types while we specifically manipulated the spatial probabilities of the target or distractor location separately and (2) examine whether differences between enhancement and suppression measures could be explained by state changes over the course of the experiment (e.g., motivation, engagement). To anticipate our results, we found the same contrast in internal reliability from Experiment 1 when using the same statistical learning task: whereas individual-difference measures of target enhancement were internally reliable, individual-difference measures of distractor suppression were not. We also found a strong correlation between Distractor Presence across TLL and DLL blocks, confirming that our contrasting results between enhancement and suppression cannot be explained by changes in states across blocks.

**Table 3:** Glossary of naming conventions for Experiments 2–5

Term	Description
**TLL **(Target-Location Learning)	Blocks in which the likelihood of the target singleton’s location is systematically manipulated.
**DLL **(Distractor-Location Learning)	Blocks in which the likelihood of the distractor singleton’s location is systematically manipulated.
**TL **(Target Location) **Frequency Effect**	The effect observed when attention is biased *toward* the likely location of the target singleton, relative to other possible target locations.
**DL** (Distractor Location) **Frequency Effect**	The effect observed when attention is biased *away from* the likely location of the distractor singleton, relative to other possible distractor locations.

### Methods

#### Participants

Forty-one undergraduate students at Johns Hopkins University participated online in this experiment for course extra credit. Participants provided informed consent, and the protocol was approved by the Johns Hopkins Homewood Institutional Review Board. As in Experiment 1, data from participants who completed the entire experiment and met performance criteria were retained. Of the 41 participants who participated, data from 13 participants were excluded from analyses due to incomplete experimental sessions. The remaining participants reported having normal or corrected-to-normal vision. Thus, data from 28 participants (mean age: 19.8 [18–23]; sex: 20 women) were retained for analysis.

#### Apparatus

The apparatus is the same as Experiment 1.

#### Stimuli and task design

**Fig. 3:**
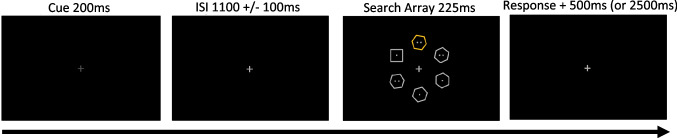
Experiment 2 Design. Similar structure to Experiment 1. Instead of letter cues, the fixation cross was dimmed to signal the start of the trial without providing information about the upcoming target array. Frequency of the target-shape-singleton location (Target-Location Learning) or distractor-color-singleton location (Distractor-Location Learning) was manipulated in separate blocks. Participants learned from experience (statistical learning) the most-probable target or distractor location, respectively

Stimuli and design are similar to Experiment 1, but with several changes (see Fig. 3). A trial began with a fixation cross that dimmed for 200 ms, serving as a ready signal. The ready signal did not provide information about the likely locations of the target or distractor, or whether a distractor would be presented in the upcoming array. A jittered delay period (1,000, 1,100, or 1,200 ms) followed the offset of the ready signal. Following the delay period, a six-item search array (visual angle radius of ~3.10°, from the fixation to the center of each display element) was presented (rather than the eight-item array as used in Experiment 1) to maintain high accuracy in the discrimination of the number of dots in the target. Visual angles of the stimuli remained the same as Experiment 1. Nontarget hexagons were limited to a rotation of 0° or 30°. An intertrial interval of 500 ms was presented following trial completion.

Target location and distractor location frequencies were manipulated in separate blocks. During trials in which a distractor was present, one of the hexagons in the array served as the distractor color singleton and displayed randomly as either orange (RGB 255, 160, 1) or green (RGB 100, 200, 49). The distractor appeared on 50% of trials for TLL blocks, balanced across target conditions (i.e., half of distractor present trials displayed the target in its frequent location, the other half of trials displayed the target in an infrequent location). Applying an analogous even-split of trials between frequent, infrequent, and distractor-absent trials, the distractor appeared on 66% of trials for DLL blocks (33% frequent-distractor-location trials, 33% infrequent-distractor-location trials). The resulting color distances (ΔE) between distractor colors and grey (RGB 169, 169, 169), the color used for all other stimuli in the array, were calculated using the CIE 1976 (L*a*b) color space (Robertson, 1977). The difference in ΔE between orange and grey (82.6272) versus green and grey (82.6159) was 0.0113.

Task procedure was similar to the Suppress-Location task in Experiment 1. In Experiment 2, there was a single task with identical instructions; only the probability of the target or distractor location was manipulated, in separate blocks. Target shape (square or diamond) and distractor color (orange or green) were randomized across trials. For TLL blocks, the target would frequently (50% probability) appear in one specific location of the array, and infrequently (~17% probability) in each of the other five locations. For DLL blocks, of the distractor-present trials, the distractor would frequently (50% probability) appear in one location of the array, and infrequently (~17% probability) in each of the other locations. The target would appear equally often in each of the five infrequent locations. Unlike Experiment 1, the target and distractor in Experiment 2 could appear at the two vertical meridian locations (12 o’clock and 6 o’clock positions). The frequent location was also the same across blocks to maximize learned frequency effects while minimizing the number of learning trials. For TLL blocks, the distractor could appear at the location of the frequent target (when the target was in an infrequent location), and for DLL blocks (as in Wang & Theeuwes, 2018b), the target could appear at the location of the frequent distractor (when the distractor was in an infrequent location).

The organization of different statistical learning block-types was adapted from Ferrante et al. (2018), where each experiment session consisted of eight experiment blocks: baseline, three learning blocks, extinction, three learning blocks, and extinction. Our baseline block consisted of 50 trials where the target and distractor locations and colors were randomized, and a distractor was present for half of the trials. The groups of three learning blocks (106 trials each) consisted of either three TLL blocks or three DLL blocks, where the frequent location remained the same across all three blocks. The frequent target and distractor locations were randomly generated. If the first group of three learning blocks was TLL, then the last group of three learning blocks was DLL and vice versa, counterbalanced across participants. The extinction blocks consisted of 100 trials where the target and distractor were present on all trials and randomly located.

#### Analysis

##### Mean differences

Similar to Experiment 1, RT and accuracy differences were compared between conditions for TLL blocks (Target Location [TL] Frequency Effect: frequent location – infrequent location; Distractor Presence Effect: distractor present, distractor absent) and DLL blocks (Distractor Location [DL] Frequency Effect: infrequent – frequent, Distractor Presence Effect: distractor present – distractor absent) using Bonferroni-corrected paired *t* tests.^2^ For both TLL and DLL blocks, trials with an RT outside of 250–2,225 ms, no response, or inappropriate key press (any key other than the right- and down-arrow response keys) were removed from accuracy analyses (1.5%). Incorrect trials were additionally removed for RT analyses (10.6%).

We applied the same difference-of-sums approach as conducted in Experiment 1 to account for individual differences in baseline RT and accuracy (i.e., Frequency Effect: (infrequent – frequent)/(infrequent + frequent), Distractor Presence Effect: (distractor present – distractor absent)/(distractor present + distractor absent)).

##### Individual differences

Reliability of individual differences was calculated using the same methods described in Experiment 1. We planned to test for correlations between the TL Frequency Effect and the DL Frequency Effect if both measures were reliable. We additionally included the Distractor Presence Effect in our individual-differences analyses to examine whether a lack of correlation between TL and DL Frequency Effects, if found, could be explained by differences in other factors (e.g., engagement, motivation) that may differ across blocks. If additional factors affect the comparison between Frequency Effects due to state changes across blocks, they would also affect the comparison of the Distractor Presence Effect.

### Results

#### Mean differences

See Table 4.

**Table 4:** Experiment 2 Normalized Pairwise Comparisons

Condition	Measurement	df	t score	p _bonferroni-corrected_	Mean % change ± se	*95% CI*	infrequent/distractor present	frequent/ distractor absent	Original (non-normalized) mean difference ± se
Enhance Location—Validity Effect									
TLL – Frequency Effect	Accuracy	27	−1.84	.15 ns	−1.3 **±** 0.7	[−2.7, 0.1]	90.0%	92.3%	−2.3% **±** 1.2%
TLL – Frequency Effect	Reaction Time	27	4.83	< .0001 ****	4.2 **±** 0.9	[2.4, 6.0]	818 ms	754 ms	64 ms **±** 13 ms
TLL – Distractor Presence	Accuracy	27	−0.47	1.00 ns	−0.2 **±** 0.4	[−0.9, 0.6]	91.0%	91.3%	−0.3% **±** 0.6%
TLL – Distractor Presence	Reaction Time	27	3.08	< .01 **	1.1 **±** 0.3	[0.4, 1.8]	793 ms	777 ms	16 ms **±** 6 ms
Suppress Location—Frequency Effect									
DLL – Frequency Effect	Accuracy	27	−2.21	.07 **.**	−0.8 **±** 0.4	[−1.6, −0.06]	89.2%	90.7%	−1.5% **±** 0.7%
DLL – Frequency Effect	Reaction Time	27	3.45	< .01 **	1.2 **±** 0.4	[0.5, 2.0]	822 ms	801 ms	21 ms **±** 6 ms
DLL – Distractor Presence	Accuracy	27	−1.95	.12 ns	−0.6 **±** 0.3	[−1.2, 0.03]	90.0%	91.0%	−1.1% **±** 0.5%
DLL – Distractor Presence	Reaction Time	27	1.72	.19 ns	0.5 **±** 0.3	[−0.09, 1.0]	811 ms	804 ms	8 ms **±** 5 ms

Mean reaction time is rounded to the nearest 1 ms and accuracy the nearest 0.1%. *ns* not significant; **.** marginal; * *p* < .05; ** *p* < .01; *** *p* < .001; **** *p* < .0001; ***** *p* < .00001

##### Reaction time

For TLL blocks, we found a significant Frequency Effect, wherein participants were faster in reporting the target when the target appeared in a frequent location than an infrequent location, *t*(27) = 4.83,* p* < .0001. We additionally found a significant Distractor Presence Effect, wherein participants were faster in reporting the target during trials where the distractor was absent than when the distractor was present, *t*(27) = 3.08,* p* < .01. For DLL blocks, we found a significant Frequency Effect wherein participants were faster in reporting the target when the distractor appeared at a frequent location than in an infrequent location,* t*(27) = 3.45,* p* < .01. There was no significant Distractor Presence Effect, *t*(27) = 1.72, *p* = .19.

##### Accuracy

Mean differences in task accuracy were consistent with the RT effects with no indication of a speed–accuracy trade-off. For TLL blocks, there was no significant Frequency Effect, *t*(27) = −1.84,* p* = .15, or Distractor Presence Effect, *t*(27) = −0.47,* p* = 1.00. For DLL blocks, we found a nonsignificant, but marginal, Frequency Effect wherein participants were more accurate in reporting the target when the distractor appeared at its frequent location than when it appeared in an infrequent location, *t*(27) = −2.21,* p* = .07. There was a nonsignificant Distractor Presence Effect, t(27) = −1.95,* p* = .12.

#### Individual differences

**Fig. 4:**
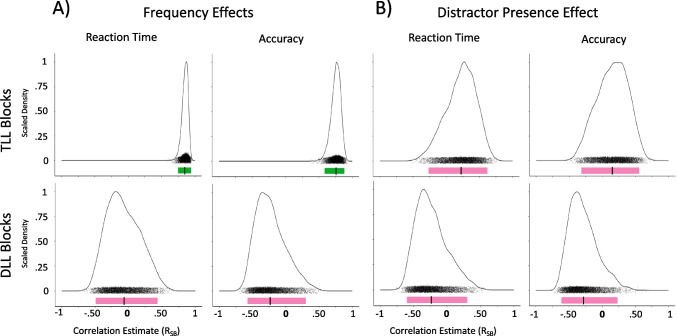
Experiment 2 density plots of Spearman Brown-corrected split half analysis with 5000 random splits (RSB). Same design procedure as Fig. 2. **A)** Frequency Effects (left two columns): Target Location (TL) Frequency Effects for Target-Location Learning (TLL) blocks (top row) and Distractor Location (DL) Frequency Effects for Distractor-Location Learning (DLL) blocks (bottom row) using reaction time (first column) and accuracy (second column). **B)** Distractor Presence Effect (right two columns) for TLL (top row) and DLL (bottom row) blocks using reaction time (third column) and accuracy (fourth column)

See Table 5 and Fig. 4.

**Table 5:** Experiment 2 Split-Half Analyses

Condition	Measurement	n	Reliability	Mean r_SB_	95% CI
Target Location Learning					
TLL – Frequency Effect	Accuracy	28	Reliable	.75	[.59, .88]
TLL – Frequency Effect	Reaction Time	28	Reliable	.85	[.75, .93]
TLL – Distractor Presence	Accuracy	28	Unreliable	.15	[-.30, .56]
TLL – Distractor Presence	Reaction Time	28	Unreliable	.23	[-.25, .62]
Distractor Location Learning					
DLL – Frequency Effect	Accuracy	28	Unreliable	-.22	[-.57, .29]
DLL – Frequency Effect	Reaction Time	28	Unreliable	-.07	[-.50, .45]
DLL – Distractor Presence	Accuracy	28	Unreliable	-.27	[-.60, .22]
DLL – Distractor Presence	Reaction Time	28	Unreliable	-.23	[-.58, .32]

##### Frequency effects

Split-half analyses showed that TLL blocks yielded internally reliable individual-difference measures of the Frequency Effect for RT, r_SB_ = 0.85, 95% CI [0.75,0.93], and accuracy, r_SB_ = 0.75, 95% CI [0.59,0.88]. DLL blocks, however, did not yield internally reliable individual-difference measures of the Frequency Effect for RT, r_SB_ = −0.07, 95% CI [−0.50,0.45], or accuracy, r_SB_ = −0.22, 95% CI [−0.57,0.29]. Due to this within-task unreliability, individual differences comparisons between enhancement strength and suppression strength would not be interpretable, and thus correlation analysis was not performed.

##### Distractor presence effect

For TLL blocks, individual-difference measures of the Distractor Presence Effect were unreliable for RT, r_SB_ = 0.23, 95% CI [−0.25, 0.62], and for accuracy, r_SB_ = 0.15, 95% CI [−0.30, 0.56]. We found that DLL blocks also yielded unreliable individual-difference measures of the Distractor Presence Effect for RT, r_SB_ = −0.23, 95% CI [−0.58, 0.32], and for accuracy, r_SB_ = −0.27, 95% CI [−0.60, 0.22].

### Conclusions

The TLL blocks yielded significant Frequency Effects at the group-level as well as internally reliable individual-difference measures. While the DLL blocks yielded significant Frequency Effects at the group-level, its individual-difference measures were unreliable. These results suggest that statistical learning itself cannot explain the lack of internal reliability in our suppression tasks (Experiments 1 and 2). One possible factor that could have affected internal reliability of the Distractor Frequency Effect in particular is if the tasks did not sufficiently constrain search strategy. Although participants were instructed to search for a target shape singleton, the target was always one of two shapes: a square or diamond. The distractor and nontargets were always hexagons. Participants could use a singleton-search strategy, which would result in attention being drawn to both the target-shape singleton and the distractor-color singleton. Participants could also utilize feature-search mode, a search strategy where features of a target would be selectively enhanced as ‘target templates’, resulting in reduced attention capture by stimuli that do not match relevant target feature(s) (Bacon & Egeth, 1994). Trial-by-trial variability in the degree to which a participant depended on singleton- versus feature-search modes may have resulted in the degree of capture by the distractor fluctuating from trial to trial, leading to less internally reliable individual-difference measures. Thus, we hypothesized that a task design that constrains participants to use only singleton-search mode might lead to better internal reliability. In Experiment 3, we changed the task design such that on every trial the shapes and colors were random, but the target was always the unique shape and the distractor was always the unique color (singleton-search task with additional singleton distractors). We examined whether internal reliability of individual differences in distractor suppression improved after constraining search strategy so that we could pursue the goal of testing for correlations between enhancement and suppression strength across participants.

## Experiment 3

Experiment 3 used a similar task as Experiment 2, but the shapes and colors of the target, the distractor, and the nontarget shapes changed randomly on every trial. Because participants could not predict target or distractor color or shape features, they could not effectively utilize feature-search mode (Bacon & Egeth, 1994). We anticipated that promoting singleton-search mode in this way would increase the degree and consistency of capture by the distractor singleton, and would, therefore, produce more internally reliable measures of individual differences in distractor suppression.

### Methods

#### Participants

Forty-six individuals participated in this online experiment for cash payment through Prolific (Prolific.co). All participants reported having normal or corrected-to-normal vision. Participants provided informed consent, and the protocol was approved by the Johns Hopkins Homewood Institutional Review Board. As in the other experiments, data from participants who completed the entire experiment and met our previously-described performance criteria were retained. Of the 46 participants who participated, the data of 19 participants were removed due to incomplete experiment sessions. The remaining participants reported having normal or corrected-to-normal vision. Thus, the data of 27 participants (mean age: 29.4 [18–39]; sex: 15 men) remained for analysis.

#### Apparatus

The apparatus is the same as Experiments 1 and 2.

#### Stimuli and task design

**Fig. 5:**
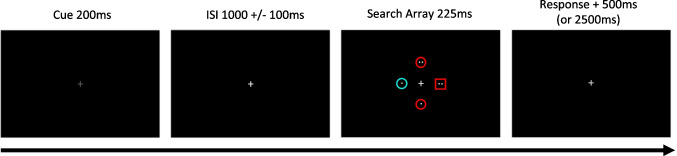
Experiment 3 Design. Same structure to Experiment 2. On every trial, the shapes and colors of the array randomly change. The target is always the unique shape singleton, and the distractor is always the unique color singleton

The task design and stimuli are similar to Experiment 2 with several notable changes (see Fig. 5). As in Experiment 2, instructions were identical for all task blocks with target location frequency manipulated in some blocks (TLL) and distractor location frequency manipulated in other blocks (DLL). One modification to the block procedure from Experiment 2 (i.e., baseline, learning blocks 1–3, extinction, learning blocks 4–6, extinction) was made for Experiment 3. The last extinction block now preceded the first set of learning blocks (i.e., Experiment 3: baseline, extinction, learning blocks 1–3, extinction, learning blocks 4–6). The purpose of the extinction blocks in both experiments was to wash out previous probability biases before beginning a new statistical learning condition. Those extinction blocks contain 100% distractor present trials so that there would be the same number of trials contributing to the extinction of distractor biases as those contributing to the extinction of target biases. This design also strengthens the consistency of potential distractor capture effects, since the probability of the presence of a distractor can also influence the strength of distractor capture (Müller et al., 2009). Additionally, the search array size was reduced from six to four items (visual angle radius of ~2.82°, from the fixation to the center of each display element) for two main reasons:

1. To maintain high accuracy for a harder task, as the random combinations of four different colors and shapes made the task more difficult, and
2. To match the number of possible shapes and colors to the number of possible locations.

Matching the number of possible shapes to locations would also facilitate use of this paradigm in future studies of feature-based statistical learning.

As noted above, we constrained participants to use singleton-search mode by changing the shapes and colors on every trial. Shapes and colors were randomly selected for each trial from a set of four shapes—circle (~1.60° × 1.60°), triangle (~1.69° × 1.50°), square (~1.50° × 1.50°), and star (~1.69° × 1.69°)—and four colors—red (RGB 255, 0, 0), magenta (RGB 255, 0, 255), green (RGB 0, 255, 0), and yellow (RGB 255, 255, 0). For each trial, the target shape was random and different from the nontarget shapes, which were homogenous. The shape of the homogenous nontargets was randomly selected from one of the remaining three shapes. The distractor color was selected at random from the set of four colors. The color of the homogenous nondistractor array items was randomly selected from one of the three remaining colors. For distractor-absent trials, all four stimuli in the array had the same color, selected at random from the pool of four colors. Lastly, in addition to randomly selecting the location of the frequent target and distractor locations for each participant as in Experiment 2, in Experiment 3 we additionally made the frequent location of the target and distractor unique for each participant. For example, if the frequent target location was top for a participant, the frequent distractor location would be any other location than the top, chosen at random.

After completing all trial blocks, participants were probed for knowledge of the high-probability target and distractor locations. Participants were first asked (Yes/No) whether they noticed any pattern in the location of the target (the unique shape on every trial). They were then told that the target did in fact appear in one location more often than the others and were asked to select the location in which they thought the target appeared most frequently (image of array was shown with the array locations marked with numbers as a reference for their selection). Participants were instructed to make their best guess if they were unsure. Participant knowledge of the frequent distractor location was then probed with the same question format. For methods and results, see Supplemental Materials Section 3.

#### Analysis

Analyses were the same as Experiment 2. For both block types, trials with an RT outside of 250–2,225 ms, no response, or inappropriate key press (any key other than the right- and down-arrow response keys) were removed from accuracy analyses (1.3%). Incorrect trials were additionally removed for RT analyses (15.7%). Analyses of the probe questions are presented in Supplemental Materials Section 3.1.1. Planned correlation and Bayes factor analyses were as described in Experiments 1 and 2.

Next, we checked whether the Frequency Effect generated during a set of statistical learning blocks (i.e., three TLL or three DLL blocks) persisted to the next block, which was the extinction block (i.e., carryover effects). If the extinction block functioned as intended, any prior Frequency Effect should have been extinguished by the end of the extinction block, prior to the next series of statistical learning blocks (i.e., no carryover effect). To test this, we coded whether the location of the target (or distractor) in a given trial was the same location previously used as the frequent location during TLL blocks. For example, if the frequent target location was presented on the left for a participant, we would code the subsequent extinction block such that any time the target were to appear on the left, the prior frequency would be labeled as ‘frequent,’ and any other location would be marked as ‘infrequent.’ We performed the same Frequency Effect analysis as described for our TLL and DLL blocks. Since TLL and DLL block order was randomized, we conducted separate analyses for participants who had previously completed TLL blocks (*n* = 15) and those who had completed DLL blocks (*n* = 12) separately.

To estimate the sensitivity of our Bayesian correlation analysis, we conducted a Monte Carlo simulation with 1,000 iterations, structured to mirror the design of our experiments. This approach allowed us to evaluate how reliably our analytic methods could detect a true correlation between individual-difference measures of enhancement and suppression, under assumptions based on our data (e.g., trial-level and subject-level variability, sample size, and trial count; for background, see Loskot, 2022; see also van Ravenzwaaij & Etz, 2021). In other words, we assessed whether our methods were sensitive enough to detect a correlation if one truly existed, given the Bayesian evidence. Given that smaller sample sizes can yield unstable estimates, we aimed to verify the robustness of our methodological approach.

For each simulation, we generated a bivariate normal distribution for latent behavioral traits, scaled using between-subject standard deviations from our TL and DL Frequency Effects. We defined an effect size threshold (i.e., true correlation estimate) using the observed correlation of Distractor Presence across TLL and DLL blocks, which are the same blocks used to compute the TL and DL Frequency Effects. Trial-level noise was incorporated by using the average within-subject standard deviation across target enhancement and distractor suppression measures, respectively. For each participant, 105 trials were simulated per condition as a conservative trial estimate. These trials were averaged and the Bayes Factor for each correlation comparison was calculated using the “correlationBF()” function in R.

### Results

#### Mean differences

See Table 6.

**Table 6:** Experiment 3 Normalized Pairwise Comparisons

Condition	Measurement	df	t score	p _bonferroni-corrected_	Mean % change ecte	*95% CI*	infrequent/distractor present	frequent/ distractor absent	Original (non-normalized) mean difference ± se
Target Location Learning									
TLL – Frequency Effect	Accuracy	26	−2.45	< .05 *	−1.7 **±** 0.7	[−3.1, −0.3]	85.1%	88.1%	−3.0% ± 1.2%
TLL – Frequency Effect	Reaction Time	26	3.64	< .01 **	2.2 **±** 0.6	[1.0, 3.4]	935 ms	897 ms	38 ms ± 11 ms
TLL – Distractor Presence	Accuracy	26	−7.32	< .00001 *****	−3.3 **±** 0.4	[−4.2, −2.4]	83.9%	89.5%	−5.6% ± 0.7%
TLL – Distractor Presence	Reaction Time	26	7.20	< .00001 *****	3.6 **±** 0.5	[2.5, 4.6]	948 ms	884 ms	65 ms ± 10 ms
Distractor Location Learning									
DLL – Frequency Effect	Accuracy	26	−0.33	1.00 ns	−0.2 **±** 0.6	[−1.4, 1.0]	84.1%	84.5%	−0.4% **±** 0.9%
DLL – Frequency Effect	Reaction Time	26	2.46	< .05 *	1.1 **±** 0.5	[0.2, 2.1]	937 ms	917 ms	20 ms **±** 10 ms
DLL – Distractor Presence	Accuracy	26	−8.91	< .00001 *****	−3.2 **±** 0.4	[−4.0, −2.5]	84.3%	89.8%	−5.5% **±** 0.6%
DLL – Distractor Presence	Reaction Time	26	5.32	< .0001 ****	2.1 **±** 0.4	[1.3, 2.9]	927 ms	888 ms	39 ms **±** 8 ms

Mean reaction time is rounded to the nearest 1 ms and accuracy the nearest 0.1%. *ns* not significant; * *p* < .05; ** *p* < .01; *** *p* < .001; **** *p* < .0001; ***** *p* < .00001

##### Reaction time

For TLL blocks, we found a significant Frequency Effect, wherein participants were faster in reporting the target during trials where the target appeared in a frequent location than an infrequent location, *t*(26) = 3.64,* p* < .01. We additionally found a significant Distractor Presence Effect, wherein participants were faster in reporting the target during trials where the distractor was absent than when the distractor was present, *t*(26) = 7.20,* p* < .00001. For DLL blocks, we found a significant Frequency Effect wherein participants were faster in reporting the target when the distractor appeared at its frequent location than when it appeared in an infrequent location, *t*(26) = 2.46,* p* < .05. We also found a significant Distractor Presence Effect wherein participants were faster in reporting the target during trials where the distractor was absent than when the distractor was present, *t*(26) = 5.32,* p* < .0001.

One of our goals in Experiment 3 was to increase the magnitude of the Distractor Presence Effect compared with Experiment 2. Compared with Experiment 2, the Distractor Presence Effect in Experiment 3 increased by 50 ms for TLL blocks and by 31 ms for DLL blocks.

##### Accuracy

Mean differences in task accuracy were consistent with the RT effects with no indication of a speed–accuracy trade-off. For TLL blocks, we found a significant Frequency Effect, wherein participants were more accurate in reporting the target when the target appeared in a frequent location than an infrequent location, t(26) = −2.45,* p* < .05. We also found a significant Distractor Presence Effect, wherein participants were more accurate in reporting the target during trials in which the distractor was absent than when the distractor was present, t(26) = −.32,* p* < .00001). For DLL blocks, we found a significant Distractor Presence Effect wherein participants were more accurate in reporting the target during trials in which the distractor was absent than when the distractor was present, *t*(26) = −8.91,* p* < .00001. There was no significant Frequency Effect, *t*(26) = −0.33,* p* = 1.00.

#### Individual differences

**Fig. 6:**
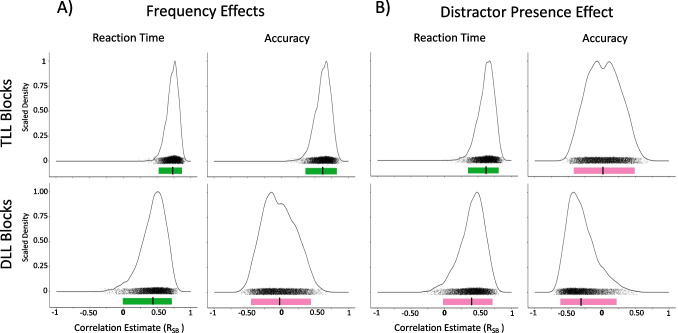
Experiment 3 density plots of Spearman Brown-corrected split half analysis with 5000 random splits (R_SB_). Same design procedure as Fig. 2. **A) **Frequency Effects (lefts two column): Target Location (TL) Frequency Effects for Target-Location Learning (TLL) blocks (top row) and Distractor Locaton (DL) Frequency Effects for Distractor-Location Learning (LL) blocks (bottom row) using reaction time (first column) and accuracy (second column). **B) **Distractor Presence Effect (right two columns) for TLL (top row) and DLL (bottom row) blocks using reaction time (third column) and accuracy (fourth column)

**Fig. 7:**
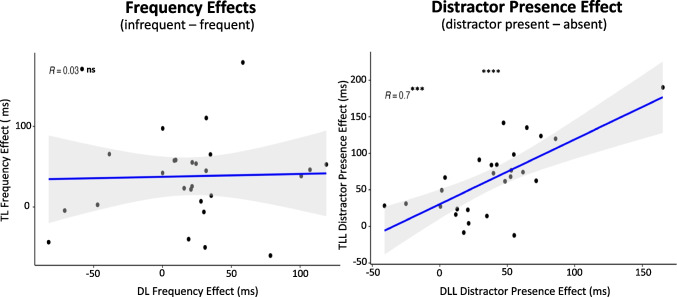
Experiment 3 comparison plots of individual-difference measures using reaction time. **Left**: comparing the Target Location (TL) Frequency Effect in Target-Location Learning (TLL) blocks and the Distractor Location (DL) Frequency Effect in Distractor-Location Learning (DLL) blocks. **Right**: comparing the Distractor Presence Effect during TLL blocks and the Distractor Presence Effect during DLL blocks

See Fig. 6 and Table 7 for split half comparisons and Fig. 7 for correlation comparisons.

**Table 7:** Experiment 3 Split-Half Analyses

condition	measurement	n	reliability	Mean r_SB_	95% CI
Target Location Learning					
TLL – Frequency Effect	Accuracy	27	Reliable	.62	[.37, .81]
TLL – Frequency Effect	Reaction Time	27	Reliable	.73	[.53, .86]
TLL – Distractor Presence	Accuracy	27	Unreliable	.02	[-.40, .49]
TLL – Distractor Presence	Reaction Time	27	Reliable	.63	[.36, .82]
Distractor Location Learning					
DLL – Frequency Effect	Accuracy	27	Unreliable	-.03	[-.44, .46]
DLL – Frequency Effect	Reaction Time	27	Reliable	.45	[.05, .73]
DLL – Distractor Presence	Accuracy	27	Unreliable	-.29	[-.62, .23]
DLL – Distractor Presence	Reaction Time	27	Unreliable	.40	[-.04, .71]

##### Frequency effects

For TLL blocks, individual-difference measures of the Frequency Effect were internally reliable for RT, r_SB_ = 0.73, 95% CI [0.53, 0.86], and accuracy, r_SB_ = 0.62, 95% CI [0.37, 0.81], as in Experiment 2. Unlike in Experiments 1 and 2, we also found that DLL blocks yielded internally reliable individual-difference measures of the Frequency Effect for RT, r_SB_ = 0.45, 95% CI [0.05, 0.73]. The Frequency Effect for accuracy remained unreliable, r_SB_ = −0.03, 95% CI [−0.44, 0.46]. As this task design yielded internally reliable individual-difference measures for RT, we were able to examine whether the magnitude of the TL Frequency Effect correlated with the magnitude of the DL Frequency Effect using RT. We found no significant correlation between the two internally reliable individual-difference measures, *t*(25) = 0.15,* p* = 1.00, *R* = 0.03. Bayesian analyses revealed anecdotal evidence in favor of the null hypothesis, BF_10_ = 0.42.

##### Distractor presence effect

For TLL blocks, we found that individual-difference measures of the Distractor Presence Effect were internally reliable for RT, r_SB_ = 0.63, 95% CI [0.36, 0.82] but not for accuracy, r_SB_ = 0.02, 95% CI [−0.40, 0.49]. For DLL blocks, we found unreliable individual-difference measures of the Distractor Presence Effect for RT, r_SB_ = 0.40, 95% CI [−0.04, 0.71], but it was near the threshold for reliability. Individual-difference measures of the Distractor Presence Effect for accuracy were unreliable, r_SB_ = −0.29, 95% CI [−0.62, 0.23].

Because the individual-differences RT measures of the Distractor Presence Effect in DLL blocks nearly met our reliability criteria, and the individual-differences RT measures of the Distractor Presence Effect in TLL blocks were internally reliable, we post hoc tested for a correlation. We found a strong and significant correlation between individual-difference measures of the Distractor Presence Effect in TLL blocks and the Distractor Presence Effect in DLL blocks using RT, *t*(25) = 4.94,* p* < .001, *R* = 0.70. Bayesian analyses revealed decisive evidence in favor of the alternative hypothesis, BF_10_ > 100.

##### Carryover effects

See Table 8. We examined whether statistical learning carryover effects were present during the extinction block immediately following a set of statistical learning blocks (i.e., TLL or DLL). For participants who first completed TLL blocks, we found no significant evidence of TL Frequency Effect carryover during the subsequent extinction block for both RT, *t*(14) = 0.60,* p* = 1.00, and accuracy, *t*(14) = 1.04,* p* = .64. For participants who first completed DLL blocks, we also found no significant evidence DL Frequency Effect carryover during the subsequent extinction block for both RT, *t*(11) = −0.60,* p* = 1.00, and accuracy, *t*(11) = −1.17,* p* = .54. These results confirm that the results from our TLL and DLL blocks are not confounded by carryover effects.

**Table 8:** Experiment 3 Normalized Pairwise Comparisons - Carry Over Effects

Condition	Measurement	df	t score	_*p*_bonferroni-corrected	Mean % change ± se	*95% CI*	infrequent/distractor present	frequent/ distractor absent	Original (non-normalized) mean difference ± se
Extinction Block – Prior Target Location Learning									
TLL – Frequency Effect	Accuracy	14	1.04	.64 ns	1.8 **±** 1.7	[−1.9, 5.4]	86.7%	84.1%	2.5% ± 2.8%
TLL – Frequency Effect	Reaction Time	14	0.60	1.00 ns	0.7 **±** 1.1	[−1.7, 3.1]	998 ms	988 ms	10 ms ± 23 ms
Extinction Block – Prior Distractor Location Learning									
DLL – Frequency Effect	Accuracy	11	−1.17	.54 ns	−1.7 **±** 1.5	[−5.0, 1.5]	84.1%	86.8%	−2.7% ± 2.4%
DLL – Frequency Effect	Reaction Time	11	−0.60	1.00 ns	−0.7 **±** 1.2	[−3.3, 1.9]	995 ms	1008 ms	−13 ms ± 23 ms

Mean reaction time is rounded to the nearest 1 ms and accuracy the nearest 0.1%. *ns* not significant; * *p* < .05; ** *p* < .01; *** *p* < .001; **** *p* < .0001; ***** *p* < .00001

##### Bayesian sensitivity simulation

In a simulation with 1,000 iterations, we defined the true correlation using our observed Distractor Presence correlation coefficient from RT, *R* = 0.70. We found that 1 of 1,000 simulations yielded a Bayes factor with our degree of evidence in favor of the null hypothesis (using a Bayes factor threshold of 0.42) given the defined true correlation. As an exploration, even if the true correlation was substantially weaker (e.g., a modest *R* = 0.20), only 52 of 1,000 yielded a Bayes factor with our degree of evidence in favor of the null hypothesis. These results provide strong support that our results of a null correlation are unlikely due to insufficient sensitivity.

### Conclusions

In Experiment 3 we found that modifying our task to encourage the use of singleton-search mode yielded internally reliable individual-difference measures of both target enhancement and distractor suppression Frequency Effects. There was no correlation, however, between TL and DL Frequency Effects. We also found internally reliable (or nearly-so) individual-difference RT measures for the Distractor Presence Effect in both TLL and DLL blocks. There was a significant correlation between the Distractor Presence Effect in TLL and DLL blocks. This significant correlation for the Distractor Presence Effect across blocks suggests that the lack of correlation between target and distractor Frequency Effects cannot be explained by state changes across blocks, such as motivation or engagement. Next, we found no evidence of carryover effects across learning blocks, suggesting that our results are not confounded from interactions between target enhancement and distractor suppression attentional biases. Additionally, our simulation findings provide strong evidence that our individual differences analyses are sufficiently sensitive. Overall, these results support the hypothesis that target enhancement and distractor suppression vary independently across individuals.

## Experiment 4

A limitation of Experiment 3 was our small sample size. We found a correlation of individual differences in Distractor Presence Effects between TLL and DLL blocks, suggesting that our methods are able to detect individual differences correlations. Thus, the goals of Experiment 4 were to test whether the results of Experiment 3 replicate to confirm our conclusions.

### Methods

#### Participants

One hundred and sixteen individuals participated in this online experiment for cash payment through Prolific. Participants provided informed consent, and the protocol was approved by the Johns Hopkins Homewood Institutional Review Board. As in the other experiments, data from participants who completed the entire experiment were retained. Of the 116 participants who participated, the data of 50 participants were removed due to incomplete experiment sessions. The remaining participants reported having normal or corrected-to-normal vision. Thus, the data of 66 participants (mean age: 28.7 [20 – 40]; sex: 33 men) remained for analysis.

#### Apparatus

The apparatus is the same as Experiment 3

#### Stimuli and task design

The task design and stimuli are the same as Experiment 3, with several notable changes to the exclusion criteria and the questionnaire process. First, participants were no longer dismissed from the study if they did not meet the previously-described performance criteria two blocks in a row. This change was made in an effort to improve reliability of our individual-difference measures by enabling a greater range of individual differences in performance. Participants were able to continue to the next block regardless of their task performance. Performance feedback was still provided at the end of each task block. Like Experiments 1–3, participants were still dismissed if they failed to follow instructions. Second, the end-of-experiment survey used in Experiments 1–3 was now divided into pre-experiment (eligibility) and postexperiment (questionnaire) segments in Experiment 4. The preexperiment segment additionally included a short color blindness screen using three color plates based on the Ishihara Color Blindness Test (Ishihara, 1964) to check for common red-green color blindness, independent of self-report. Participants needed to correctly report two of the three color plates to pass our color blindness screening. While the Prolific recruitment process was designed to screen for eligibility, the preexperiment questionnaire was included to confirm eligibility. Individuals whose responses indicated that they were not eligible (e.g., under 18 years or not normal color vision) would have been dismissed from the study (none needed to be excluded based on answers to the questions). Some sections of the postexperiment questionnaire that were not directly relevant to our hypotheses were eliminated to reduce total experiment duration. Lastly, the order of the probe questions was randomized, such that some participants were probed regarding the frequent target location first, and others the frequent distractor location first. This was done to control for possible order effects. For results of the probe, see Supplemental Materials Section 4.

#### Analysis

Analyses were the same as Experiment 3. For both block types, trials with an RT outside of 250–2,225 ms, no response, or inappropriate key press (any key other than the right- and down-arrow response keys) were removed from accuracy analyses (1.3%). Incorrect trials were additionally removed for RT analyses (18.2%).

### Results

#### Mean differences

See Table 9.

**Table 9:** Experiment 4 Normalized Pairwise Comparisons

Condition	Measurement	df	t score	p _bonferroni-corrected_	Mean % change ecte	*95% CI*	infrequent/distractor present	frequent/ distractor absent	Original (non-normalized) mean difference ± se
Target Location Learning									
TLL – Frequency Effect	Accuracy	65	−3.40	< .01 **	−1.4 **±** 0.4	[−2.2, −0.6]	83.3%	85.6%	−2.3% ± 0.7%
TLL – Frequency Effect	Reaction Time	65	5.49	< .00001 *****	2.1 **±** 0.4	[1.3, 2.8]	877 ms	841 ms	36 ms ± 6 ms
TLL – Distractor Presence	Accuracy	65	−10.89	< .00001 *****	−4.9 **±** 0.4	[−5.8, −4.0]	80.4%	88.6%	−8.2% ± 0.7%
TLL – Distractor Presence	Reaction Time	65	11.86	< .00001 *****	3.8 **±** 0.3	[3.1, 4.4]	892 ms	827 ms	65 ± 5 ms
Distractor Location Learning									
DLL – Frequency Effect	Accuracy	65	−2.25	.06 **.**	−1.3 **±** 0.6	[−2.4, −0.1]	80.6%	82.6%	−2.0% **±** 0.9%
DLL – Frequency Effect	Reaction Time	65	2.16	.07 **.**	0.6 **±** 0.3	[0.04, 1.2]	895 ms	885 ms	11 ms **±** 5 ms
DLL – Distractor Presence	Accuracy	65	−9.00	< .00001 *****	−3.2 **±** 0.4	[−4.0, −2.5]	81.6%	87.0%	−5.4% **±** 0.6%
DLL – Distractor Presence	Reaction Time	65	10.57	< .00001 *****	3.2 **±** 0.3	[2.6, 3.8]	889 ms	832 ms	57 ms **±** 6 ms

Mean reaction time is rounded to the nearest 1 ms and accuracy the nearest 0.1%. *ns* not significant; **.** marginal; * *p* < .05; ** *p* < .01; *** *p* < .001; **** *p* < .0001; ***** *p* < .00001

##### Reaction time

For TLL blocks, we found a significant Frequency Effect, wherein participants were faster in reporting the target when the target appeared in a frequent location than an infrequent location, *t*(65) = 5.49,* p* < .00001. We additionally found a significant Distractor Presence Effect, wherein participants were faster in reporting the target during trials when the distractor was absent than when the distractor was present, *t*(65) = 11.86,* p* < .00001. For DLL blocks, there was a nonsignificant, but marginal, Frequency Effect, wherein participants were faster in reporting the target when the distractor appeared at a frequent location than an infrequent location, t(65) = 2.16,* p* = .07. We found a significant Distractor Presence Effect, wherein participants were faster in reporting the target during trials when the distractor was absent than when the distractor was present, *t*(65) = 10.57,* p* < .00001.

##### Accuracy

Mean differences in task accuracy were consistent with the RT effects with no indication of a speed–accuracy trade-off. For TLL blocks, we found a significant Frequency Effect, wherein participants were more accurate in reporting the target when the target appeared in a frequent location than an infrequent location, *t*(65) = −3.40,* p* < .01. We also found a significant Distractor Presence Effect, wherein participants were more accurate in reporting the target during trials where the distractor was absent than when the distractor was present, *t*(65) = −10.89,* p* < .00001. For DLL blocks, we found a nonsignificant, but marginal, Frequency Effect, wherein participants were more accurate in reporting the target when the distractor appeared in a frequent location than an infrequent location, *t*(65) = −2.25,* p* = .06. We found a significant Distractor Presence Effect, wherein participants were more accurate in reporting the target during trials when the distractor was absent than when the distractor was present, *t*(65) = −9.00,* p* < .00001.

#### Individual differences

**Fig. 8:**
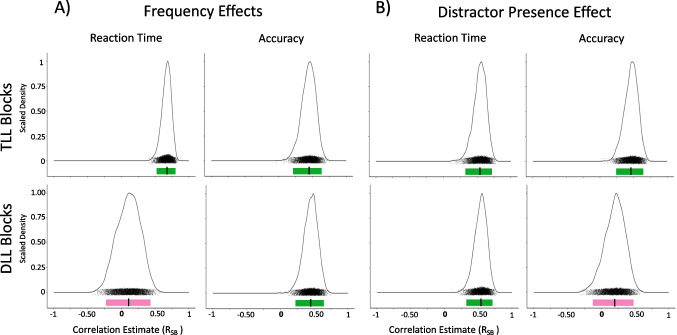
Experiment 4 density plots of Spearman Brown-corrected split half analysis with 5000 random splits (R_SB_). Same design procedure as Fig. 2. **A**) Frequency Effects (left two columns): Target Location (TL) Frequency Effects for Target-Location Learning (TLL) blocks (top row) and Distractor Location (DL) Frequency Effects for Distractor-Location Learning (DLL) blocks (bottom row) using reaction time (first and accuracy (second column). **B)** Distractor Presence Effect (right two columns) for TLL (top row) and DLL (bottom row) blocks using time (third column) and accuracy (fourth column)

**Fig. 9:**
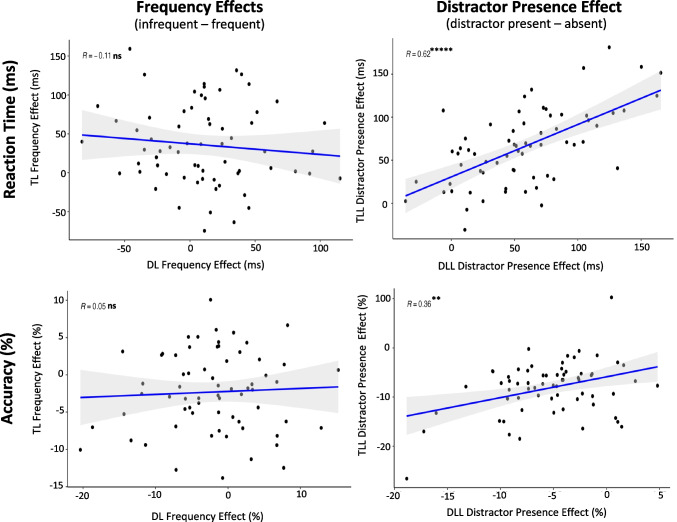
Experiment 4 comparison plots of individual-difference measures using reaction time (top row) and accuracy (bottom row). **Left column: **comparing the Target Location (TL) Frequency Effect in Target-Location Learning (TLL) blocks and the Distractor Location (DL) Frequency Effect in Distractor-Location Learning (DLL) blocks. **Right column: **comparing the Distractor Presence Effect during TLL blocks and the Distractor Presence Effect during DLL blocks

See Table 10 and Fig. 8 for split half comparisons and Fig. 9 for correlation comparisons.

**Table 10:** Experiment 4 Split-Half Analyses

Condition	Measurement	n	Reliability	Mean r_SB_	95% CI
Target Location Learning					
TLL – Frequency Effect	Accuracy	66	Reliable	.44	[.21, .63]
TLL – Frequency Effect	Reaction Time	66	Reliable	.67	[.52, .79]
TLL – Distractor Presence	Accuracy	66	Reliable	.46	[.25, .64]
TLL – Distractor Presence	Reaction Time	66	Reliable	.54	[.33, .71]
Distractor Location Learning					
DLL – Frequency Effect	Accuracy	66	Reliable	.45	[.23, .64]
DLL – Frequency Effect	Reaction Time	66	Unreliable	.11	[-.23, .42]
DLL – Distractor Presence	Accuracy	66	Unreliable	.20	[-.12, .47]
DLL – Distractor Presence	Reaction Time	66	Reliable	.54	[.33, .70]

##### Frequency effects

Split-half analyses showed that, for TLL blocks, the Frequency Effect yielded internally reliable individual differences for RT, r_SB_ = 0.67, 95% CI [0.52 0.79], and accuracy, r_SB_ = 0.44, 95% CI [0.21 0.63]. For DLL blocks, individual differences of the Frequency Effect were reliable for accuracy, r_SB_ = 0.45, 95% CI [0.23, 0.64], but not for RT, r_SB_ = 0.11, 95% CI [−0.23, 0.42]. As this task design yielded internally reliable individual-difference measures of both TL and DL Frequency Effects for accuracy, we were able to examine whether the two measures correlated. We found no significant correlation between the two internally reliable individual-difference measures of accuracy, *t*(64) = 0.42,* p* = 1.00, *R* = 0.05, with substantial evidence in favor of the null hypothesis, BF_10_ = 0.30.

Because this was a replication study, we also tested for a correlation between individual differences in the TL and DL Frequency Effects using RT despite the lack of internal reliability in this measure. We found no significant correlation between the two individual-difference measures, *t*(64) = −0.87,* p* = .77, *R* = −0.11, with anecdotal evidence in favor of the null hypothesis, BF_10_ = 0.40.

##### Distractor presence effect

For TLL blocks, the Distractor Presence Effect yielded internally reliable individual differences for RT, r_SB_ = 0.54, 95% CI [0.33, 0.71] and accuracy, r_SB_ = 0.46, 95% CI [0.25, 0.64]. DLL blocks also yielded internally reliable individual differences of the Distractor Presence Effect for RT, r_SB_ = 0.54, 95% CI [0.33, 0.70], but not for accuracy, r_SB_ = 0.20, 95% CI [−0.12, 0.47]. As this task design yielded internally reliable individual-difference measures of the Distractor Presence Effect for RT, we were able to examine whether the Distractor Presence Effect correlated across TLL and DLL blocks. We found a significant correlation between the two internally reliable individual-difference measures for RT, *t*(64) = 6.36,* p* < .00001, *R* = 0.62, with decisive evidence in favor of the alternative hypothesis, BF_10_ > 100.

Because this was a replication study, we also tested for a correlation between individual differences in the Distractor Presence Effect for TLL blocks and the Distractor Presence Effect for DLL blocks using accuracy, despite the lack of internal reliability for DLL blocks. We found a significant correlation between the two individual-difference measures, *t*(64) = 3.12,* p* < .01, *R* = 0.36, with very strong evidence in favor of the alternative hypothesis, BF_10_ = 17.94.

##### Carryover effects

See Table 11. For participants who first completed TLL blocks, we found no significant Frequency Effect during the proceeding extinction block for RT, *t*(34) = 2.11,* p* = .08. For participants who first completed DLL blocks, we found no significant Frequency Effect during the proceeding extinction block for RT, *t*(30) = −0.73,* p* = .94, and accuracy, *t*(30) = −0.45,* p* = 1.00. We did find, however, a significant Frequency Effect during the proceeding extinction block for accuracy for participants who first completed TLL blocks, *t*(34) = −3.11,* p* < .01. To verify whether, and for how long, the TL Frequency Effect persisted, we checked for carryover effects during the next block immediately following extinction, which was the first of three DLL blocks. We examined the first half of trials from the first DLL block (~ 50 trials) to verify whether the previous TL Frequency Effect was extinguished early on in the DLL block set.^3^ We found that the TL Frequency Effect from the previous TLL blocks were no longer significant within the first half of the first DLL block, *t*(34) = −2.10,* p* = .09. In other words, any potential carryover effects from our previous TLL manipulation were fully extinguished within the first 16% of DLL trials. These results confirm that our statistical learning effects are not contaminated from carry over effects.

**Table 11:** Experiment 4 Normalized Pairwise Comparisons - Carry Over Effects

Condition	Measurement	df	t score	p _bonferroni-corrected_	Mean % change ecte	95% CI	infrequent/distractor present	frequent/ distractor absent	Original (non-normalized) mean difference ± se
Extinction Block – Prior Target Location Learning									
TLL – Frequency Effect	Accuracy	34	−3.11	< .01 **	−3.1 **±** 1.0	[−5.1, 1.1]	80.0%	85.3%	−5.3% ± 1.6%
TLL – Frequency Effect	Reaction Time	34	2.11	.08 ns	1.2 **±** 0.6	[0.05, 2.5]	932 ms	910 ms	22 ms ± 11 ms
First half of first DLL block immediately following extinction – Prior Target Location Learning									
TLL – Frequency Effect	Accuracy	34	−2.10	.09 ns	−3.1 **±** 1.5	[−6.2, −0.09]	83.4%	85.7%	−5.3% ± 2.3%
Extinction Block – Prior Distractor Location Learning									
DLL – Frequency Effect	Accuracy	30	−0.45	1.00 ns	−0.6 **±** 1.3	[−3.2, 2.0]	82.3%	83.6%	−1.4% ± 1.9%
DLL – Frequency Effect	Reaction Time	30	−0.73	.94 ns	−0.5 **±** 0.6	[−1.8, 0.8]	955 ms	970 ms	−15 ms ± 13 ms

Mean reaction time is rounded to the nearest 1 ms and accuracy the nearest 0.1%. *ns* not significant; * *p* < .05; ** *p* < .01; *** *p* < .001; **** *p* < .0001; ***** *p* < .00001

##### Bayesian sensitivity simulation

In a simulation with 1,000 iterations, we defined the true correlation using our observed Distractor Presence correlation coefficient from RT, *R* = 0.63, and from accuracy,* R* = 0.36. For both RT and accuracy, 0 of 1,000 RT simulations and 0 of 1,000 accuracy simulations yielded a Bayes factor with our degree of evidence in favor of the null hypothesis (using thresholds of 0.27 and 0.24, respectively) given the defined true correlation. As an exploration, even if the true correlation was substantially weaker (e.g., a modest *R* = 0.20, 0 of 1,000 RT simulations and 0 of 1,000 accuracy simulations yielded a Bayes factor with our degree of evidence in favor of the null hypothesis, respectively). These results provide strong support that our results of a null correlation are unlikely a result of insufficient sensitivity to detect a true correlation.

#### Post hoc analysis of Experiments 3 and 4 combined

In addition to our present findings, we performed an additional post hoc analysis by combining Experiments 3 and 4 data to examine our effects using a larger sample size (*N* = 93).

##### Frequency effects

The combined datasets of Experiment 3 and 4 yielded internally reliable individual-difference measures for both TL Frequency Effects—RT: r_SB_ = 0.68, 95% CI [0.56, 0.78], accuracy: r_SB_ = 0.50, 95% CI [0.32, 0.64]—and DL Frequency Effects—nearly so for RT: r_SB_ = 0.23, 95% CI [−0.05, 0.46], accuracy: r_SB_ = 0.37, 95% CI [0.16, 0.54]. We found no significant correlation between individual-difference measures of TL and DL Frequency Effects—RT: *t*(91) = −0.55,* p* = .59, *R* = −0.06, accuracy: *t*(91) = 0.21,* p* = .84, *R* = 0.02—with substantial evidence in favor of the null hypothesis—RT: BF_10_ = 0.27, accuracy: BF_10_ = 0.24.

##### Distractor presence effect

We also found internally reliable individual-difference measures for the Distractor Presence Effect during TLL blocks—RT: r_SB_ = 0.56, 95% CI [0.41, 0.69], accuracy: r_SB_ = 0.41, 95% CI [0.21, 0.58]—and DLL blocks for RT, r_SB_ = 0.52, 95% CI [0.34, 0.66]; individual-difference measures for accuracy were unreliable, r_SB_ = 0.08, 95% CI [−0.19, 0.33].

Because this was a replication study, we tested for correlations in both RT and accuracy despite the weak internal reliability for accuracy. We found a significant correlation between individual-difference measures of the Distractor Presence Effect during TLL and DLL blocks—RT: *t*(91) = 7.74,* p* < .00001, *R* = 0.63, accuracy: *t*(91) = 3.19,* p* < .01, *R* = 0.32—with decisive and very strong evidence in favor of the alternative hypothesis—RT: BF_10_ > 100, accuracy: BF_10_ = 21.89, respectively.

### Conclusions

In general, the results of Experiment 4 confirmed the conclusions of Experiments 2 and 3. Measures of target enhancement were again internally reliable. Measures of distractor suppression continued to be less reliable than those of target enhancement although they were far more reliable in both Experiments 3 and 4, in which participants were constrained to a singleton-search strategy, than in Experiment 2. The measure of suppression that did show internal reliability in Experiment 4 (DL Frequency Effect for accuracy) did not correlate with the corresponding measure of enhancement (TL Frequency Effect for accuracy). Bayesian analyses indicated evidence in favor of the null hypothesis. These results are similar to the findings in Experiment 3, except they are for accuracy instead of RT. When the datasets of Experiments 3 and 4 were combined, we again found no correlations between suppression and enhancement and stronger evidence in favor of the null hypothesis in Bayesian analyses. Therefore, the results of Experiment 4 strengthened our conclusions that individual differences in target enhancement are largely independent of individual differences in distractor suppression, as measured by TL and DL Frequency Effects following statistical learning. The results support the hypothesis that enhancement and suppression mechanisms are separable.

Experiment 4 also confirmed the conclusions of Experiment 3, indicating the lack of correlation is specific to our comparison of enhancement and suppression measures and cannot be explained by differences in engagement, motivation, or other factors that could have varied across task blocks. For Experiment 4 and when datasets from Experiments 3 and 4 were combined, both TLL and DLL blocks yielded internally reliable individual-difference measures for the Distractor Presence Effect. These internally reliable measures did significantly correlate with each other, with strong Bayesian evidence in favor of the alternative hypothesis. Any general performance factors that could have differed between the TLL and DLL blocks would have affected the comparisons of the Distractor Presence Effect measures as well as the Frequency Effects. In addition, like Experiment 3, the simulation findings of Experiment 4 provide strong evidence that our individual differences analyses are sufficiently sensitive. Thus, the presence of a strong correlation between the TLL Distractor Presence Effect and the DLL Distractor Presence Effect strengthens the conclusion that the lack of a correlation between the TLL and DLL Frequency Effects reflects a true null result.

Finally, in Experiment 5, at the suggestion of a reviewer, we replicated Experiments 3 and 4 in a larger cohort.

## Experiment 5

Although our replication and simulations provide strong evidence for a dissociation between measures of target enhancement and distractor suppression, we recognize that readers may remain cautious about conclusions drawn from modest sample sizes. Small sample sizes can be unstable and can lead to inaccurate conclusions (Schönbrodt & Perugini, 2013), exacerbating the current crisis of replication. To address such potential concerns, in Experiment 5, we conducted an additional study with a larger sample size approaching 250 participants, consistent with stability estimations from Schönbrodt and Perugini (2013). Thus, in Experiment 5 we sought to replicate our experimental methods to see whether our conclusions from Experiments 3 and 4 may be verified with a larger sample size.

### Methods

#### Participants

Two-hundred and fifty three individuals participated in this online experiment for cash payment through Prolific. Participants provided informed consent, and the protocol was approved by the Johns Hopkins Homewood Institutional Review Board. As in the other experiments, data from participants who completed the entire experiment were retained. Of the 253 participants who participated, the data of 54 participants were removed due to incomplete experiment sessions. The remaining participants reported having normal or corrected-to-normal vision. Thus, the data of 199 participants (mean age: 29.5 [18–41]; sex: 101 men) remained for analysis.

#### Apparatus

The apparatus is the same as Experiment 3 and Experiment 4.

#### Stimuli and task design

The task design and stimuli are the same as Experiment 4, with a minor change. Previously, the number of dots in the distractor stimulus were always the opposite the number in the target stimulus. This method was intended to exclude participants who may have been responding to the salient distractor rather than the target. In Experiment 5, however, we instead randomized the number of dots in the distractor relative to the target. This change was made to reduce the risk that participants could deduce the number of target dots without paying attention to the target (i.e., always report the number of dots opposite the distractor).

#### Analysis

Analyses were the same as Experiment 4. For both block types, trials with an RT outside of 250–2,225 ms, no response, or inappropriate key press (any key other than the right- and down-arrow response keys) were removed from accuracy analyses (1.75%). Incorrect trials were additionally removed for RT analyses (25.77%).

### Results

#### Mean differences

See Table 12.

**Table 12:** Experiment 5 Normalized Pairwise Comparisons

Condition	Measurement	df	t score	p _bonferroni-corrected_	Mean % change ± se	*95% CI*	infrequent/distractor present	frequent/ distractor absent	Original (non-normalized) mean difference ± se
Target Location Learning									
TLL – Frequency Effect	Accuracy	198	−4.82	< .00001 *****	−1.7 **±** 0.4	[−2.4, −1.0]	75.5%	78.1%	−2.6% ± 0.5%
TLL – Frequency Effect	Reaction Time	198	7.01	< .00001 *****	1.5 **±** 0.2	[1.1, 1.9]	969 ms	942 ms	27 ms ± 4 ms
TLL – Distractor Presence	Accuracy	198	−8.77	< .00001 *****	−2.3 **±** 0.3	[−2.8, −1.8]	75.1%	78.5%	−3.4% ± 0.4%
TLL – Distractor Presence	Reaction Time	198	12.84	< .00001 *****	2.2 **±** 0.2	[1.9, 2.6]	977 ms	934 ms	43 ms ± 3 ms
Distractor Location Learning									
DLL – Frequency Effect	Accuracy	198	−0.60	1.00 ns	−0.2 **±** 0.3	[−0.9, 0.4]	74.3%	74.6%	−0.3% **±** 0.5%
DLL – Frequency Effect	Reaction Time	198	−0.29	1.00 ns	−0.05 **±** 0.2	[−0.4, 0.3]	970 ms	972 ms	−1 ms **±** 3 ms
DLL – Distractor Presence	Accuracy	198	−7.98	< .00001 *****	−2.0 **±** 0.3	[−2.5, −1.5]	74.5%	77.6%	−3.1% **±** 0.4%
DLL – Distractor Presence	Reaction Time	198	12.58	< .00001 *****	2.1 **±** 0.2	[1.8, 2.4]	971 ms	932 ms	40 ms **±** 3 ms

Mean reaction time is rounded to the nearest 1 ms and accuracy the nearest 0.1%. *ns* not significant; * *p* < .05; ** *p* < .01; *** *p* < .001; **** *p* < .0001; ***** *p* < .00001

##### Reaction time

For TLL blocks, we found a significant Frequency Effect, wherein participants were faster in reporting the target when the target appeared in a frequent location than an infrequent location, *t*(198) = 7.01,* p* < .00001. We additionally found a significant Distractor Presence Effect, wherein participants were faster in reporting the target during trials when the distractor was absent than when the distractor was present, *t*(198) = 12.84,* p* < .00001. For DLL blocks, there was a nonsignificant Frequency Effect, t(198) = −0.29,* p* = 1.00. We found a significant Distractor Presence Effect, wherein participants were faster in reporting the target during trials when the distractor was absent than when the distractor was present, *t*(198) = 12.58,* p* < .00001.

##### Accuracy

Mean differences in task accuracy were consistent with the RT effects with no indication of a speed–accuracy trade-off. For TLL blocks, we found a significant Frequency Effect, wherein participants were more accurate in reporting the target when the target appeared in a frequent location than an infrequent location, *t*(198) = −4.82,* p* < .00001. We also found a significant Distractor Presence Effect, wherein participants were more accurate in reporting the target during trials where the distractor was absent than when the distractor was present, *t*(198) = −8.77,* p* < .00001. For DLL blocks, we found a nonsignificant Frequency Effect, t(198) = −0.60,* p* = 1.00. We next found a significant Distractor Presence Effect, wherein participants were more accurate in reporting the target during trials when the distractor was absent than when the distractor was present, *t*(198) = −7.98,* p* < .00001.

#### Individual differences

**Fig. 10:**
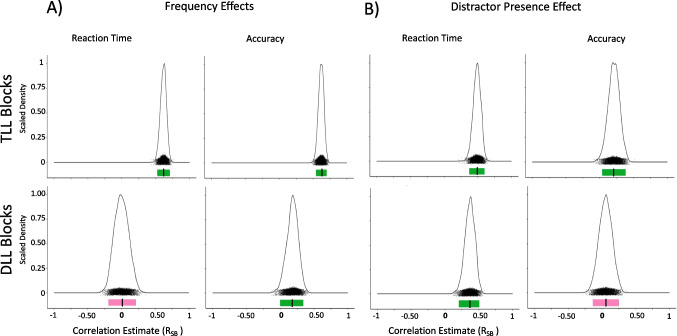
Experiment 5 density plots of Spearman Brown-corrected split half analysis with 5000 random splits (R_SB_). **A**) Frequency Effects columns (left two columns): Target Location (TL) Frequency Effects for Target-Location Learning (TLL) blocks (top row) and Distractor Location (DL) Frequency Effects for Distractor-Location Learning (DLL) blocks (bottom row) using reaction time (first column) and accuracy (second column). **B**) Distractor Presence Effect (right two columns) for TLL (top row) and DLL (bottom row) blocks using reaction time (third column) and accuracy (fourth column)

**Fig. 11:**
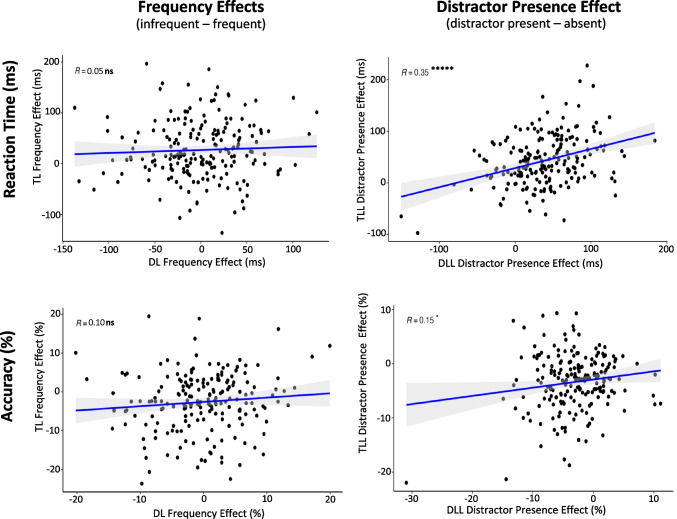
Experiment 5 comparison plots of individual-difference measures using reaction time (top row) and accuracy (bottom row). **Left column:** comparing the Target Location (TL) Frequency Effect in Target-Location Learning (TLL) blocks and the Distractor Location (DL) Frequency Effect in Distractor-Location Learning (DLL) blocks. **Right column: **comparing the Distractor Presence Effect during TLL blocks and the Distractor Presence Effect during DLL blocks

See Fig. 10 and Table 13 for split half comparisons and Fig. 11 for correlation comparisons.

**Table 13:** Experiment 5 Split-Half Analyses

Condition	Measurement	n	Reliability	Mean r_SB_	95% CI
Target Location Learning					
TLL – Frequency Effect	Accuracy	199	Reliable	.62	[.53, .69]
TLL – Frequency Effect	Reaction Time	199	Reliable	.61	[.52, .69]
TLL – Distractor Presence	Accuracy	199	Reliable	.21	[.04, .37]
TLL – Distractor Presence	Reaction Time	199	Reliable	.49	[.37, .59]
Distractor Location Learning					
DLL – Frequency Effect	Accuracy	199	Reliable	.18	[.01, .34]
DLL – Frequency Effect	Reaction Time	199	Unreliable	.00	[-.19, .20]
DLL – Distractor Presence	Accuracy	199	Unreliable	.05	[-.13, .24]
DLL – Distractor Presence	Reaction Time	199	Reliable	.37	[.21, .50]

##### Frequency effects

Split-half analyses showed that, for TLL blocks, the Frequency Effect yielded internally reliable individual differences for RT, r_SB_ = 0.61, 95% CI [0.52 0.69], and accuracy, r_SB_ = 0.62, 95% CI [0.53 0.69]. For DLL blocks, individual differences of the Frequency Effect were reliable for accuracy, r_SB_ = 0.18, 95% CI [0.01, 0.34], but not for RT, r_SB_ = 0.00, 95% CI [−0.19, 0.20]. As this task design yielded internally reliable individual-difference measures of both TL and DL Frequency Effects for accuracy, we were able to examine whether the two measures correlated. We found no significant correlation between the two internally reliable individual-difference measures of accuracy, *t*(197) = 1.37,* p* = .35, *R* = 0.10, with anecdotal evidence in favor of the null hypothesis, BF_10_ = 0.41.

Because this was a replication study, we also tested for a correlation between individual differences in the TL and DL Frequency Effects using RT despite the lack of internal reliability in this measure. We found no significant correlation between the two individual-difference measures, *t*(197) = 0.66,* p* = 1.00, *R* = 0.05, with substantial evidence in favor of the null hypothesis, BF_10_ = 0.20.

##### Distractor presence effect

For TLL blocks, the Distractor Presence Effect yielded internally reliable individual differences for RT, r_SB_ = 0.49, 95% CI [0.37, 0.59] and accuracy, r_SB_ = 0.21, 95% CI [0.04, 0.37]. DLL blocks again yielded internally reliable individual differences of the Distractor Presence Effect for RT, r_SB_ = 0.37, 95% CI [0.21, 0.50], but not for accuracy, r_SB_ = 0.05, 95% CI [−0.13, 0.24]. As this task design yielded internally reliable individual-difference measures of the Distractor Presence Effect for RT, we were again able to examine whether the Distractor Presence Effect correlated across TLL and DLL blocks. We found a significant correlation between the two internally reliable individual-difference measures for RT, *t*(197) = 5.28,* p* < .00001, *R* = 0.35, with decisive evidence in favor of the alternative hypothesis, BF_10_ > 100.

Because this was a replication study, we also tested for a correlation between individual differences in the Distractor Presence Effect for TLL blocks and the Distractor Presence Effect for DLL blocks using accuracy, despite the lack of internal reliability for DLL blocks. We found a nonsignificant (but trending) correlation between the two individual-difference measures, *t*(197) = 2.08,* p* = .08, *R* = 0.15, with anecdotal evidence in favor of the alternative hypothesis, BF_10_ = 1.33.

As a result of maximizing the magnitude of our individual-difference measures, the magnitude of our group difference measures were consequently statistically minimized (Hedge et al., 2018). Although we suspect that participants with negative suppression difference measures are likely following task instructions, we aimed to rule out, post-hoc, whether the lack of covariance found between enhancement and suppression measures could be explained by including negative Frequency Effect measures. Thus, we performed a correlation analysis that only including participants with a suppression effect greater than 0 for RT (*N* = 99) and less than 0 for accuracy (*N* = 100). We again found no significant correlation between measures of accuracy, *t*(98) = 0.37,* p* = 1.00, *R* = 0.04, with substantial evidence in favor of the null hypothesis, BF_10_ = 0.25. Individual-difference measures of RT again did not covary, *t*(97) = 0.34,* p* = 1.00, *R* = 0.03, with substantial evidence in favor of the null hypothesis, BF_10_ = 0.24.

##### Carryover effects

See Table 14 for results. For participants who first completed TLL blocks, we found no significant TL Frequency Effect during the subsequent extinction block for RT, *t*(109) = −1.30,* p* = .39, and accuracy, *t*(109) = 2.04,* p* = .09. For participants who first completed DLL blocks, we found no significant DL Frequency Effect during the subsequent extinction block for and RT, *t*(88) = −0.18,* p* = 1.00, and accuracy: *t*(88) = −0.70,* p* = .97. These results confirm that our analyses of TLL and DLL blocks are not confounded by carryover effects.

**Table 14:** Experiment 5 Normalized Pairwise Comparisons - Carry Over Effects

Condition	Measurement	df	t score	p _bonferroni-corrected_	Mean % change ecte	*95% CI*	infrequent/distractor present	frequent/ distractor absent	Original (non-normalized) mean difference ± se
Extinction Block – Prior Target Location Learning									
TLL – Frequency Effect	Accuracy	109	2.04	.09 ns	1.5 **±** 0.7	[0.4, 2.9]	77.0%	75.3%	1.8% ± 1.0%
TLL – Frequency Effect	Reaction Time	109	−1.30	.39 ns	−0.5 **±** 0.4	[−1.3, 0.3]	1005 ms	1018 ms	−12 ms ± 8 ms
Extinction Block – Prior Distractor Location Learning									
DLL – Frequency Effect	Accuracy	88	−0.70	.97 ns	−0.5 **±** 0.7	[−1.9, 0.9]	76.6%	77.5%	−1.0% ± 1.0%
DLL – Frequency Effect	Reaction Time	88	−0.18	1.00 ns	−0.08 **±** 0.4	[−0.9, 0.8]	1001 ms	1003 ms	−2 ms ± 9 ms

Mean reaction time is rounded to the nearest 1 ms and accuracy the nearest 0.1%. *ns* not significant; * *p* < .05; ** *p* < .01; *** *p* < .001; **** *p* < .0001; ***** *p* < .00001

##### Bayesian sensitivity simulation

In a simulation with 1,000 iterations, we defined the true correlation using our observed Distractor Presence correlation coefficient from RT, *R* = 0.35, and from accuracy, *R* = 0.36. For both RT and accuracy, 1 of 1000 simulations for RT and 7 of 1,000 simulations for accuracy yielded a Bayes Factor with our degree of evidence in favor of the null hypothesis (using thresholds of 0.20 and 0.41, respectively) given the defined true correlation. As an exploration, if the true correlation was substantially weaker (e.g., a modest *R* = 0.20), 43 of 1,000 using RT measures and 218 of 1,000 simulations using accuracy measures yielded a Bayes factor with our degree of evidence in favor of the null hypothesis, respectively. These results support that our methods are sufficiently sensitive to detect a true correlation if it were present.

### Conclusions

Experiment 5 serves as an additional replication to support the results of Experiments 3 and 4 in a larger sample. Measures of target enhancement were again internally reliable. Measures of distractor suppression continued to be less reliable than those of target enhancement, although they were more reliable in Experiments 3, 4, and 5, in which participants were constrained to a singleton-search strategy, than in Experiments 1 and 2. The measure of suppression that did yield internal reliability in Experiment 4 (DL Frequency Effect for accuracy) was replicated in Experiment 5 and did not correlate with the corresponding measure of enhancement (TL Frequency Effect for accuracy). Bayesian evidence was also in favor of the null hypothesis.

Furthermore, our simulation findings of Experiment 5 provide convergent evidence that our individual differences analyses are sufficiently sensitive and replicate the sensitivity findings of Experiments 3 and 4. Irrespective of the size of our samples, the probability of yielding our Bayesian evidence in favor of the null hypothesis, given that an empirically-estimated true correlation does exist, is highly unlikely. Therefore, the results of Experiment 5—in combination with the results of Experiments 3 and 4—provide strong, convergent, support for our conclusions that individual differences in target enhancement are largely independent of individual differences in distractor suppression, as measured by TL and DL Frequency Effects following statistical learning. The results strongly support the hypothesis that enhancement and suppression mechanisms are separable.

## General discussion

The ability to focus on relevant target information and ignore irrelevant distracting information is crucial in navigating through a world saturated with information, yet it is not yet fully understood what mechanisms underlie target enhancement and distractor suppression. The same behavior (e.g., difficulties completing an attention-demanding task) could result from impairments in different neural mechanisms of attention (e.g., difficulty enhancing targets versus difficulty suppressing distractors) which might require different interventions. Studies have shown, for example, evidence of changes in the N2pc and P_D_ components in children with attention-deficit/hyperactivity disorder (ADHD) compared with neurotypical children (Wang et al., 2016). If enhancement and suppression mechanisms are at least partially separable, then having reliable, dissociable measures of individual differences in enhancement and suppression will be critical in improving outcomes for these children. Such measures could be used to improve diagnostic accuracy and therapeutic efficacy. Understanding how enhancement and suppression may vary independently across individuals may also enable the amelioration of many issues of public health concern, such as occupational and driving safety. In support of these goals, the present study used an individual differences approach to address whether separable mechanisms of enhancement and suppression exist. Here we presented a series of five experiments that utilized attention paradigms adapted from methods introduced by Posner (1980) and Wang and Theeuwes (2018b) to investigate whether individual differences in enhancement and suppression strengths are internally reliable, and if so, whether the magnitude of enhancement strength correlates with the magnitude of suppression strength.

The results highlight a striking contrast in internal reliability between enhancement and suppression; while enhancement is consistently internally reliable across tasks and search strategies, suppression is variable and sensitive to task parameters, consistent with previous research (see Sauter et al., 2018; Zhang et al., 2019). In other words, these results suggest that the ways in which enhancement and suppression are implemented across contexts and strategies differ. Examining individual-difference measures, particularly for suppression tasks, has proven challenging in the field in part because of this variability across trials (Rouder et al., 2023). Our experimental design included as many trials per condition as was feasible while maintaining participant engagement. Across experiments, we adjusted our task design to maximize internal reliability. In Experiments 3, 4, and 5, our within-session reliability was clearly sufficient to find strong correlations when they exist (i.e., Distractor Presence measures strongly correlated across Target- and Distractor-Location Learning blocks). Furthermore, when enhancement and suppression both yielded internally reliable individual differences in Experiments 3, 4, and 5, they did not significantly correlate. These results signify that any mechanistic relationship between enhancement and suppression (at least via statistical learning of target and distractor locations) is weak, and shared mechanisms cannot fully explain their underlying processes. Increasing the magnitude of distractor capture did not change these results. Our findings directly contradict the notion that enhancement and suppression are simply two aspects of the same underlying neural mechanism of attention. Instead, our results provide convergent support for the existence of separable neural mechanisms of target enhancement and distractor suppression.

### Sources of variability

In the current study, we considered the mechanisms of enhancement and suppression to be “separable” if there was evidence for distinct sources of variance for these two processes across individuals. Variance across individuals could arise from stable factors (i.e., genetic, developmental, or life-long experience-based factors) or potentially dynamic factors (i.e., strategy, physiological state changes, contextual changes, recent experience).

#### Cellular circuitry

If the neural mechanisms of enhancement and suppression were both parts of a single integrated, interdependent, cellular circuit then these factors affecting interindividual variance would be expected to have similar, or at least correlated, effects on enhancement and suppression. If the neural mechanisms were clearly separate, however, relying on different brain structures, circuitry, or neurotransmitters, for example, then a factor resulting in differences across individuals (such as a genetic variant or a difference in arousal) could affect enhancement efficacy without affecting suppression efficacy or vice versa.

However, there are also multiple ways in which enhancement and suppression could vary somewhat independently across individuals, but still be considered the result of a “shared” neural mechanism. Independent variability could indicate that the mechanisms are not identical or symmetrical, but they could still be inter-related. For example, a correlation could be weakened by distinct individual differences in the properties of the shape, size, or sensitivities of excitatory receptive fields that are different from those of inhibitory receptive fields, even though the excitatory and inhibitory processes are both part of the same circuit. In this case, increased excitatory gain would be predicted to lead to increased inputs to lateral inhibition. The impact of these inputs on suppression, however, might vary across individuals independently of inter-individual variability in excitatory gain (see Ni et al., 2012, for an analogous example of variability in asymmetry across neurons). Such neural properties, however, would be consistent within individuals, which would still predict a correlation between enhancement and suppression, even if weak. Our results, however, show no significant evidence for a correlation between enhancement and suppression, suggesting more separability than merely independent variability in gain for enhancement versus suppression within parts of an interdependent circuit.

#### Search strategies

Activation of mechanisms underlying distractor suppression may be more dependent on the particular search strategy deployed than are the mechanisms of enhancement. We show internal reliability for individual differences in measures of enhancement, for both cue validity effects and learned frequency effects. Individual-difference measures for suppression, however, were consistently unreliable until search strategy was strongly constrained to singleton-search mode (Experiments 3 and 4 and 5). This interpretation, that suppression is more sensitive to strategy than it is to enhancement, is supported by previous studies that found strategy can affect behavior in search tasks with salient distractors (e.g., Bacon & Egeth, 1994; Folk et al., 2002; Kerzel & Huynh Cong, 2022). To this effect, it was possible in our study to use multiple search strategies explicitly in Experiment 2 (e.g., feature-search mode and singleton-search mode) before we constrained search strategy to singleton-search mode in Experiments 3 and 4 and 5. In fact, had participants only been using singleton-search mode in Experiment 2, our results of Experiment 2 should have closely aligned with the results of Experiments 3 and 4 and 5, which we did not observe. Thus, the additional option of feature-search mode as a viable strategy may have affected the underlying mechanisms of distractor suppression. The deployment of an attentional template (via feature-search mode) would result in selectively enhancing stimuli that match the attentional template (e.g., square or diamond target) and perhaps relatively suppressing stimuli outside of this template, including the distractors (e.g., nontarget hexagons). Indeed, the Distractor Presence Effect was smaller, on average, in Experiment 2 than in Experiments 3 and 4 and 5, in which singleton-search mode was required for successful performance. The smaller Distractor Presence Effect in Experiment 2 suggests less attention to the distractors, which is consistent with previous studies showing that during feature search mode, distractors can be suppressed even below baseline (Gaspelin et al., 2015). Decreased attention to the distractors could have also resulted in less statistical learning and smaller Distractor Location Frequency Effects on average (Baker et al., 2004; but see Musz et al., 2015). Future research is needed to understand how search strategies differently affect enhancement versus suppression.

#### Effects of experience

In addition to the effects of search strategy, recent experience might also have contributed to inconsistencies in distractor effects across trials. While target enhancement can be produced by a cue alone, previous studies have shown that suppression can be deployed reactively, which requires first selection followed by inhibition of the distractor location (Moher & Egeth, 2012), or requires repeated experience (Vatterott & Vecera, 2012), and cannot be produced solely by cueing a feature (Cunningham & Egeth, 2016; Stilwell & Vecera, 2019). Although it has been shown that distractor cues can improve target search by proactively implementing a ‘template for rejection’ (Arita et al., 2012; Chidharom & Carlisle, 2023), attempts to replicate this effect have found conflicting results. Some studies find that successful use of a distractor cue is possible but is contextual and sensitive to search strategy (Becker et al., 2015; Xu et al., 2023), while others find no evidence of a proactive deployment of suppression (Addleman & Störmer, 2022; for review see Geng, 2014). Stilwell and Vecera (2019) show that interference caused by distractor feature cues can be attenuated to baseline with prior experience of the distractor feature.

In addition, suppression has been shown to fluctuate rapidly with changes in distractor probabilities (Moher et al., 2011; see also Blais et al., 2012). Thus, in our experiments, suppression could have varied across trials due to random fluctuations in the percentage of the immediately-preceding trials in which the distractors appeared in the frequent location. Enhancement, as indicated by its greater internal reliability, appears to be less sensitive to such recent history effects. These additional differences between enhancement and suppression regarding internal reliability and dependence on strategy and recent experience are additional evidence for mechanisms that are at least partially distinct.

#### States versus traits

Sources of variability across individuals for either enhancement or suppression could be either trait-like (varying very slowly across time and generally stable across days) or state-like (varying much more quickly, such as across days or even across trials within an experimental session). Independent variability for enhancement versus suppression could indicate separable mechanisms regardless of the time scale. Our findings from Experiments 1 and 2 suggest that individual differences in enhancement are stable across a longer time scale (at least within an experimental session) than are individual differences in suppression (which frequently vary across trials), providing additional support for the hypothesis that separable mechanisms exist. These differences in the timescales of variability could suggest differences in the effects of neuromodulators on circuits involved in enhancement versus those involved in suppression (Thiele & Bellgrove, 2018). Interestingly, the magnitude of our Distractor-Location Frequency Effect in Experiment 1 was comparable to that of our Target-Location Frequency Effects in Experiments 2–4, yet the individual-difference measures of suppression were entirely unreliable. This suggests that our unreliable suppression measures cannot be sufficiently explained simply by a weak effect. When we did find reliable individual-difference measures for both enhancement and suppression in Experiments 3 and 4, the internal reliability of enhancement was still clearly stronger than that of suppression. Although internal reliability for suppression measures improved, these results suggest that suppression may always be more variable across trials than enhancement, regardless of the search strategy implemented.

The internal reliability of individual differences in enhancement across all four of our experiments does suggest that the factors driving these differences do not vary much across trials within an experimental session. Individual differences in suppression, however, did seem to be driven in part by factors (such as search strategy) that could vary rapidly, including across trials within a session (see review by Irons & Leber, 2020). This rapid variability could explain the lack of internal reliability observed in Experiments 1 and 2. Such apparent differences in the time course of variability for enhancement versus suppression is additional evidence for separable mechanisms. Future research examining the time course of variations in suppression measures within and across individuals is needed. This future research should include examining test–retest reliability across days using a statistical learning paradigm, which would provide a stronger indication of trait-like individual differences.

### Future directions

#### Physiological predictions

A remaining question is what the neural bases of these separable mechanisms are. For example, it remains unknown whether ERP components that have previously been shown to be related to enhancement (N2pc and N_T_) and suppression (P_D_) reflect the independent sources of variance identified in our study. It would be powerful evidence in favor of independent neural mechanisms if individual differences in these physiological measures do not correlate, as we found for behavioral measures. These ERP measures, or other EEG or fMRI measures, of individual differences in enhancement and suppression might also enable identification of distinct neural architectures for enhancement versus suppression.

#### Implications for models of attention

The biased-competition and normalization models of attention appear, from previous research, to apply well to the tuning of population codes (simultaneously increasing gain in some neurons and decreasing it in others) to optimize representation only of relevant information following attentional selection (e.g., reviewed in Reynolds & Heeger, 2009). Such a mechanism may underlie shared variance in selective enhancement of attended stimuli and generalized decreases in the processing of unattended stimuli (reviews in Chelazzi et al., 2019; Noonan et al., 2018). These models do not, however, appear to account well for individual differences in the selection and discrimination of targets and the suppression of salient distractors in visual search tasks, as they do not predict the results of the current study or those of similar studies in the literature. Accounting for both sets of results may improve model applicability across a greater variety of tasks and real-world scenarios.

## Conclusion

In conclusion, the individual differences approach presented here enabled a dissociation of the selective enhancement and selective suppression components affecting attention-related behavior. The results provide strong support for separable mechanisms for target enhancement and distractor suppression based on differences in internal reliability and uncorrelated individual differences when the corresponding measures are both internally reliable. Future research is needed to determine the neural bases for individual differences in target enhancement and for individual differences in distractor suppression using functional and structural imaging.

## Supplementary Information

Below is the link to the electronic supplementary material.

ESM 1 (DOCX 23.8 KB)

## Footnotes

1 Although a 3 × 1 repeated-measures ANOVA with Greenhouse–Geisser correction (`Greenhouse & Geisser, 1959) using the “ezANOVA()” function from the R package *ez* (Version 4.4.0; `Lawrence, 2016) was initially conducted to include a neutral baseline for comparison (i.e., non-informative cues for Enhance-Location, distractor absent for Suppress-Location), these reports are not reported in this article due to concerns of their interpretability from the low proportion of these types of trials; such design nuances may result in inconsistent and incorrect baseline interpretations (Jonides & Mack, 1984).

2 Similar to Experiment 1, we originally performed ANOVAs in Experiment 2. To maintain manuscript clarity, we present in the main text the results of our normalized *t* tests. Our original ANOVA tests, which include statistics for possible interactions between conditions, can be found Supplemental Materials Section 2 for reference.

3 We intended to compare the first half to the second half of the extinction block to examine whether any potential carry-over effects were extinguished by the end of the extinction block, but we found that there were too few trials per frequent condition to reliably measure a prior Frequency Effect (e.g., often less than 10 frequent trials per participant). Thus, we decided instead to examine the following DLL block, which is the first DLL block of three. For DLL blocks, the first half still included more than 30 frequent trials per participant.
