# Supplementary material for: Not simply two sides of the same coin: Target enhancement and distractor suppression draw on independent neural mechanisms
Source: Atten Percept Psychophys. 2026 Apr 6;88(4):101. doi: 10.3758/s13414-025-03196-2 (PMC13053411; doi:10.3758/s13414-025-03196-2)
Supplement: Supplementary file 1 — (DOCX 23.8 KB) [file 13414_2025_3196_MOESM1_ESM.docx]

**Supplementary Materials**

**1. Experiment 1**

**1.1 Methods**

***1.1.1. Task, stimuli, and procedure***

During the Enhance-Feature task, participants were cued to the likely shape of the target. The cue correctly predicted the target shape (Enhance-Feature) with 70% validity (“S” or “D” for square or diamond target, respectively; equal probabilities of S vs. D). The cue invalidly predicted the target shape in 20% of trials (32 trials). The “N” letter cue did not provide any information about the upcoming target for 10% of trials (16 trials). Targets did not appear at the two vertical meridian indexes (12 o’clock and 6 o’clock positions), as our Enhance-Location task only cued to the right or left side.

During the Suppress-Feature task, the distractor singleton would have one particular color with 70% probability (Frequent trials) and any of the four other colors at random with 20% probability (Infrequent trials); distractor singleton location was random. Neither targets nor distractor-singletons appeared at the two vertical meridian indexes (12 o’clock and 6 o’clock positions), as our Enhance-Location task only cued to the right or left side.

***1.1.2. Questionnaire***

Participants were provided a questionnaire at the end of the experiment which took roughly 15 minutes to complete. Participants were asked about demographics, education and skills, sex, gender, vision and hearing, health history, the Alcohol Use Disorders Identification Test (AUDIT; Saunders et al., 1993), the Barkley Adult ADHD Rating Scale (BAARS-IV; Barkley, 2011), cardiovascular and respiratory fitness (McAuley et al., 2011), the Fagerstrom Nicotine Dependence Test (Heatherton et al., 1991), the Perceived Stress Scale (Cohen et al., 1983), and the Pittsburgh Sleep Quality Index (Buysse et al., 1989).

**2. Experiment 2**

**ANOVA Methods**

For TLL blocks, accuracy and RT were analyzed using a 2x2 repeated measures ANOVA (Target Location [TL] Frequency Effect: frequent location, infrequent location; Distractor Presence Effect: distractor present, distractor absent). An analogous procedure was utilized for DLL blocks with a 3x1 repeated measures ANOVA (Distractor Condition: frequent location, infrequent location, distractor absent), with Greenhouse-Geisser correction. Difference scores were then compared between conditions (Distractor Location [DL] Frequency Effect: infrequent – frequent, Distractor Presence Effect: distractor present – distractor absent) using Bonferroni-corrected paired t-tests.

**3. Experiment 3**

**3.1. Analysis**

***3.1.1. Statistical Learning Probe***

Probe responses were contrasted and compared to chance: 50% for the first Yes/No question, and 25% for the location identification questions since there were four possible locations. Pairwise comparisons were conducted to examine whether each response category – (1) No + Incorrect Response, (2) No + Correct Reponses, (3) Yes + Incorrect Response, (4) Yes + Correct Response – was significantly different from chance-level.

***3.2.1. Statistical Learning Probe***

We found that 96% of all participants reported being unaware of any pattern in the location of the target singleton. Although these participants reported a lack of awareness, 57.7% of the unaware participants reported the correct high probability target location, significantly above chance, 25%; t(26) = 3.1 p < 0.01. 78% of all participants reported being unaware of any pattern in the location of the distractor singleton. Only 24% of the unaware participants reported the correct high probability distractor location, below what would be expected by chance, t(26) = -0.85 p = 0.40. These results of a lack of conscious awareness suggest that participants learned both target- and distractor-frequency effects via statistical learning rather than explicit pattern-solving. Our target frequency probe results also suggest that, even though participants reported a lack of awareness of the high-probability target location, they still possessed some degree of retained and reportable information guiding their correct probe selection. Participants were not able to similarly use learned information about the frequent location of distractors to guide probe selection.

**4. Experiment 4**

***4.1.1. Statistical Learning Probe***

We found that 86% of all participants reported being unaware of any pattern in the location of the target singleton. Although these participants reported a lack of awareness, 42% of the unaware participants reported the correct high probability target location. This was non-significantly (but marginally) above chance of 25%, t(65) = 1.90 p = 0.06. We found that 76% of all participants reported being unaware of any pattern in the location of the distractor singleton. Although 40% of the unaware participants reported the correct high probability distractor location, this was not significantly above chance, t(65) = 0.93, p = 0.36. These results are consistent with our findings from 3.2.1.

**References**

Barkley, R. A. (2011). Barkley Adult ADHD Rating Scale-IV (BAARS-IV). The Guilford Press.

Buysse, D. J., Reynolds III, C. F., Monk, T. H., Berman, S. R., & Kupfer, D. J. (1989). The Pittsburgh Sleep Quality Index: a new instrument for psychiatric practice and research. Psychiatry Research, 28(2), 193–213.

Cohen, S., Kamarck, T., & Mermelstein, R. (1983). A global measure of perceived stress. Journal of health and social behavior, 24(4), 385–396.

Heatherton, T. F., Kozlowski, L. T., Frecker, R. C., & Fagerström, K. O. (1991). The Fagerström Test for Nicotine Dependence: a revision of the Fagerström Tolerance Questionnaire. *British journal of addiction*, *86*(9), 1119–1127. <https://doi.org/10.1111/j.1360-0443.1991.tb01879.x>

McAuley, E., Szabo, A. N., Mailey, E. L., Erickson, K. I., Voss, M., White, S. M., Wójcicki, T. R., Gothe, N., Olson, E. A., Mullen, S. P., & Kramer, A. F. (2011). Non-Exercise Estimated Cardiorespiratory Fitness: Associations with Brain Structure, Cognition, and Memory Complaints in Older Adults. Mental health and physical activity, 4(1), 5–11. <https://doi.org/10.1016/j.mhpa.2011.01.001>

Saunders, J. B., Aasland, O. G., Babor, T. F., De la Fuente, J. R., & Grant, M. (1993). Development of the alcohol use disorders identification test (AUDIT): WHO collaborative project on early detection of persons with harmful alcohol consumption‐II. Addiction, 88(6), 791-804.
